# Temporal Characteristics of Gustatory Responses in Rat Parabrachial Neurons Vary by Stimulus and Chemosensitive Neuron Type

**DOI:** 10.1371/journal.pone.0076828

**Published:** 2013-10-04

**Authors:** Laura Geran, Susan Travers

**Affiliations:** Division of Oral Biology, College of Dentistry, the Ohio State University, Columbus, Ohio, United States of America; University of Tokyo, Japan

## Abstract

It has been demonstrated that temporal features of spike trains can increase the amount of information available for gustatory processing. However, the nature of these temporal characteristics and their relationship to different taste qualities and neuron types are not well-defined. The present study analyzed the time course of taste responses from parabrachial (PBN) neurons elicited by multiple applications of “sweet” (sucrose), “salty” (NaCl), “sour” (citric acid), and “bitter” (quinine and cycloheximide) stimuli in an acute preparation. Time course varied significantly by taste stimulus and best-stimulus classification. Across neurons, the ensemble code for the three electrolytes was similar initially but quinine diverged from NaCl and acid during the second 500ms of stimulation and all four qualities became distinct just after 1s. This temporal evolution was reflected in significantly broader tuning during the initial response. Metric space analyses of quality discrimination by individual neurons showed that increases in information *(H*) afforded by temporal factors was usually explained by differences in rate envelope, which had a greater impact during the initial 2s (22.5% increase in *H*) compared to the later response (9.5%). Moreover, timing had a differential impact according to cell type, with between-quality discrimination in neurons activated maximally by NaCl or citric acid most affected. Timing was also found to dramatically improve within-quality discrimination (80% increase in *H*) in neurons that responded optimally to bitter stimuli (B-best). Spikes from B-best neurons were also more likely to occur in bursts. These findings suggest that among PBN taste neurons, time-dependent increases in mutual information can arise from stimulus- and neuron-specific differences in response envelope during the initial dynamic period. A stable rate code predominates in later epochs.

## Introduction

Distinctive temporal variations in gustatory responses have been noted since the earliest single-unit studies in the taste system (e.g., [1,2,3]). Over the years, interest in these aspects of the response has waxed and waned but until recently, investigators addressing gustatory quality coding have typically utilized measures that sum spikes over several seconds. In the last decade, an emphasis on temporal phenomena has re-emerged, facilitated in part by advancements in computational power, electrophysiology and analytical methods that can rigorously assess how time contributes to taste coding (e.g., [4,5]).

A series of investigations in the first-order taste relay, the nucleus of the solitary tract (NST), used metric space analysis to demonstrate that temporal factors can increase the amount of mutual information that single neurons convey about taste quality, particularly in broadly-tuned neurons [5,6,7,8]. A recent study suggests that a similar temporal enhancement also occurs downstream in the parabrachial nucleus (PBN; [9]). Although these findings provide evidence that the temporal features of brainstem taste responses have the capacity to augment quality coding, neither the nature of those temporal features nor how they relate to different taste qualities or chemosensitive neuron types is well-defined. The current study used metric space and other temporal analyses to better define several time-varying response features of PBN neurons, as well as their relationships to each other.

The PBN is a complex region that in rodents forms an obligatory relay for conveying information via the thalamocortical pathway and sends projections to homeostatic regions of the ventral forebrain [10]. Our previous investigation of this nucleus emphasized heterogeneity in response profiles, including a novel group of cells that responded selectively to bitter stimuli [11]. However, responses were characterized only by summed spike measures. The current results demonstrate that the time course of taste-elicited PBN responses is complex and depends upon stimulus and chemosensitive neuron type. The initial response period is highly dynamic, coinciding with rapid changes in the ensemble code and a greater degree of temporal enhancement of mutual information in single neurons. As in our previous investigation, we observed neurons optimally responsive to bitter stimuli that received input from the palate and posterior papillae but not the anterior tongue; the present analyses revealed that these neurons had a distinctive temporal signature. Moreover, the contribution of temporal factors to between-quality discrimination was more limited for these cells than for neurons optimally responsive to electrolytes, but made a notable contribution to discrimination between bitter tastants.

## Methods

### Surgery

All experimental protocols were approved by the Ohio State University Institutional Animal Care and Use Committee in accordance with guidelines from the National Institutes of Health. Adult male Sprague-Dawley rats were anesthetized with an initial dose of thiobutabarbital (100 mg/kg Inactin i.p.) and maintained at a surgical plane with supplementary doses of sodium pentobarbital (15 mg/kg Nembutal i.p.). A heating pad and thermistor were used to keep body temperature at approximately 37°C. Sutures were threaded through the lips to keep the mouth open and a fine suture passed just under the mucosa anterior to the foliate papillae so that they could be gently stretched to facilitate stimulus access. Bleeding was negligible and no obvious inflammation occurred. A midline incision was made in the ventral neck, and the superior laryngeal and hypoglossal nerves bilaterally transected with microscissors. The trachea was then exposed and a small plastic tube inserted and tied into place to aid breathing. The incision was closed with wound clips and the animal placed in a stereotaxic head holder. A second incision was made on the top of the skull and the skin and fascia removed to expose lambda. A small hole was drilled into the parietal bone lateral to the midline and anterior to the transverse sinus, and the dura removed.

### Data collection

A low impedance search electrode (0.2 to 1.0 MOhm, FHC Inc.) was slowly driven into the brain with a microdrive (Kopf) at 1.8 mm lateral and 0.2 mm anterior to lambda. This was done at an angle 20° anterior to perpendicular in order to avoid the transverse sinus. An audio monitor was used to find the appropriate landmarks and their depths recorded in a laboratory notebook. Taste-responsive areas were found by alternately infusing the oral cavity with distilled water and a search mixture containing representatives of the 4 taste qualities. All stimuli, including distilled water, were kept at room temperature. The electrode was considered to be at or near the waist region of PBN [12] when the response to the taste mixture exceeded the response to water over a depth of 400-500µm. Once the waist was located, the electrode was replaced with a higher impedance tungsten electrode (2.0 to 4.0 MOhms, WPI Inc.) and the computer-controlled flow system pressurized and oriented such that the taste solutions contacted the entire oral cavity. Single neuron taste responses were then isolated during repeated stimulus trials and receptive field testing. Signal to noise ratios exceeded 3:1 and template matching (Spike 2) was performed to make sure all action potentials were from the same cell. The receptive field test was performed by stroking a small paintbrush dipped in distilled water over a given papillary field, followed by one dipped in the taste mixture. Fields tested include the anterior tongue (AT), nasoincisor ducts (NID), soft palate including geschsmackstreiffen (SP), and foliate papillae (FOL). In a subset of cases, lesions (3-5µA/3s) were made at or near the location where a neuron had been recorded. At the conclusion of the experiment, animals were injected with 150mg/kg of sodium pentobarbital and then perfused with saline, followed by formalin. Subsequently, brains were extracted and stored in sucrose-formalin until they were sectioned at 52µm and mounted as alternate series. Each series was stained with cresyl violet or Weil to reveal soma and myelinated fibers, respectively. Sections were inspected with a light microscope (Nikon E600) and photomicrographs taken of sections containing lesions.

### Stimuli

Each stimulus trial consisted of 5 phases: 10s spontaneous activity, 10s distilled water, 10s tastant, 20s distilled water rinse and another 10s of spontaneous activity. The fluid flowed continuously from one phase to the next so that any phasic responses to the tactile and thermal components of the stimulus were adapted before taste onset. Intertrial intervals lasted at least 1 minute and all fluids were delivered at a rate of 2 ml/s. Five stimuli were used to determine the chemosensitive response profile of each neuron; 0.1 M NaCl (“salty”), 0.3 M sucrose (“sweet”), 0.03 M quinine HCl (“bitter”), 0.03 M citric acid (“sour”) and 10 µM cycloheximide (“bitter”). Two bitter stimuli were used because we have found cycloheximide to be particularly effective in stimulating bitter-best neurons in the past [11,13], and quinine is the bitter stimulus most commonly used in the literature. In order to robustly activate bitter-sensitive cells regardless of best stimulus type, we used a concentration of quinine 1 log step higher than the concentration used in our previous investigations. Whenever possible, we also tested additional stimuli representing the most effective quality for each neuron (e.g. 0.02 M Na saccharin, “sweet”; 0.01 M HCl, “sour”) to assess within-quality discrimination.

### Data analysis

#### Basic response measures and response envelopes

The main data set consisted of 29 neurons tested with stimuli representing each of the 4 taste qualities. Trials of all 5 stimuli were repeated several times for each cell in order to assess temporal coding. The 5 stimuli were repeated 3-12 times (mean + s.e. = 7.4 + 0.47, mode = 10); 25 of 29 neurons received at least 5 repetitions of each stimulus. In a number of cases an additional trial was completed for the majority of stimuli. There was no significant correlation between the number of trials and the contribution of time to mutual information (H_temp_) for any of the three time periods analyzed (R’s = -0.01-.216; P’ s 0.26-0.99).

Spikes were sorted and counted for each phase of every trial using Spike 2 software (CED Ltd.). Similar to our previous studies [11,13], the basic measure used to characterize response profiles for a neuron was the net number of spikes in the 10s taste period after subtracting activity occurring during the preceding water stimulation. Responses were then averaged across trials. To determine whether a stimulus elicited a significant response in a given cell, paired t-tests were used to compare firing rate during water and tastant stimulation across multiple trials. To measure the variability of responses across trials, the coefficient of variation (CV = SD/mean) was calculated for the 10s net responses for each stimulus eliciting a significant response in a given cell. Breadth of tuning was characterized by two measures: entropy (BOT_H_ = -K ∑P_i (1, n)_ log P_i_ [14]) and noise-to-signal ratio (N:S, see [15]). The noise-to-signal ratio was defined as the mean of the response to the second-best stimulus divided by the mean of the response to the best stimulus [15]. Neurons were divided into groups via hierarchical cluster analysis using the 10s summed responses. Similarity between neuron profiles was defined based on Pearson’s r and profiles were amalgamated with the method of average linkage (Systat, v. 13). The number of groups was determined by examining the scree plot for abrupt jumps in amalgamation distance. Response envelopes over time were characterized using net firing rates in successive 100ms bins. Average response envelopes were first generated across different trials of the same stimulus for a given cell. Mean response envelopes for different stimuli were then calculated across the entire population of cells and by neuron type. Net summed activity for the first second, first 2s, and middle 2s (seconds 5-7) of the responses was subsequently calculated to generate chemosensitive profiles that could be compared across different time points.

#### Metric Space Analysis

For each neuron, spike times from the 10s taste period for each trial were used to populate an array. This array was tested for evidence of temporal contribution using the spike train analysis (STA) toolkit for Matlab [16]. As explained in detail in earlier publications, including several by the Di Lorenzo laboratory using data from taste-responsive cells, metric space analysis first derives distances between spike trains elicited by different stimuli and trials for a given neuron [5,6,7,8,16]. These distances were calculated in two ways: 1) on the basis of spike count alone (i.e. rate coding), where the distance between two spike trains is simply the difference between the numbers of spikes and 2) by taking the temporal structure of the spike trains into account, where the distance is the minimum distance between two spike trains calculated on the basis of matching both the temporal structure and numbers of spikes as closely as possible by moving spikes in time as well as adding or deleting them. Because the resolution of the putative temporal code is unknown, the impact of temporal structure was tested at several different levels of precision, “*q*”, where *q* is in units of 1/s, and q*t is the “cost” of moving a spike a certain distance in time, “t”. When *q*=t, the cost of shifting a spike in time is equal to adding or removing one. As in several previous studies using metric space analysis in the brainstem [5,6,7,8,16], *q* ranged from 0.0625-500, where 1/*q* = 16-0.002s; thus, temporal precision encompassed time scales ranging from seconds to milliseconds. The distances for spike count (*q*=0) and each value of *q* were subsequently used to predict the stimulus associated with a given spike train and the resulting confusion matrices (i.e., predicted versus observed stimulus) were the basis for calculating mutual information for each level of *q* and for spike rate alone (*H*_count_). Mutual information, or *H*, is measured in bits, so the maximum amount of information possible for 2 stimuli is 1 (or log_2_ 2) while the maximum possible for 4 stimuli is 2 (or log_2_ 4). The maximum amount of mutual information for each comparison is denoted *H*_max_ and the corresponding *q* value labeled *q*_max_. Two sets of controls were performed for the metric analyses. In the “shuffling” control, stimulus labels were assigned randomly to the spike trains to simulate chance. The “exchange” control, on the other hand, interchanged the spike times among trials for the same stimulus thus changing the precise times at which spikes occurred while preserving the overall temporal pattern, or rate envelope, of the response. If the *H*_max_ of the original data exceeded the *H*_max_ over all values of the exchange control, this indicated that the advantage conferred by time had a contribution from precise spike timing and not from rate envelope alone. Forty exchanges were performed for each comparison [5].

In the first set of metric space analyses, we analyzed responses to the four “standard” stimuli (sucrose, NaCl, citric acid and quinine) to determine the impact of temporal factors on between-quality discrimination. For this analysis, cycloheximide was eliminated so that the stimulus array was comparable to earlier metric space analyses [5,9]. To determine whether temporal contributions differed according to the epoch analyzed, four different time periods were considered: the entire 10s, the first s, the first 2s and the middle 2s. The first 2s and entire 10s periods were used so that our data could be compared to earlier studies. The first s and the middle 2s were also included to capture the most dynamic and stable periods of the response. Analysis of the early response periods (i.e. first s and 2s) began 0.5s after valve opening to compensate for the lag necessary for the stimulus to contact the mouth (measured lag: X=611 + 89ms, n=55); the middle period analyzed was from 25-27s, starting halfway through the taste period. In addition, we also used metric space analyses to determine the impact of temporal factors in discriminating between stimuli of a single quality. Most of these analyses focused on quinine and cycloheximide, but additional stimulus pairs were available as described in the Results section. These within-quality analyses utilized the entire 10s period because many of the neurons for which within-quality data were available included longer-latency, slowly incrementing responses to bitter stimuli.

#### Bursting Analysis

During the course of data collection, we noticed that neurons optimally responsive to bitter stimuli tended to respond with repeated bursts of action potentials. Thus, a final set of analyses was carried out to systematically quantify bursting. Data was drawn from a larger sample of neurons (n=122, including 5 additional cells from the current study that were not used in the metric analyses due to a low number of repeated trials and 88 from a previous PBN investigation (see [11])). Responses were taken from the first trial for a given stimulus and included all significant responses elicited by: 300mM sucrose (n=18), 30 mM monopotassium glutamate + 3 mM inosine monophosphate (n=10), 100mM NaCl (n=79), 10 mM HCl (n=11), 30 mM citric acid, (n=54), 30 mM quinine-HCl(n=28), and 0.01mM cycloheximide (n=30). The last 5s of the stimulation period was analyzed to avoid confounds introduced by fluctuations in the phasic portion of the response and to accommodate the slowly-incrementing responses often elicited by bitter stimuli. Bursts were defined based upon the “Poisson Surprise” method developed by Legendy and Salcman [17] and used successfully in a variety of neural systems [18,19,20]. This method assigns elevations in spike rate a “surprise” value, “S”= -logP, where P is the probability that a local increment in activity would occur at a given mean firing rate with a Poisson distribution. We set the criterion for a burst at S = 3, corresponding to a 5% probability. This metric was implemented using a script available from CED for Spike 2 (“surprise. s2s”). Identified bursts were analyzed further using a second script (“Burst. s2s”, v. 1.31), which yielded additional measures including burst duration and within-burst interspike interval (ISI) as well as the percent of spikes in bursts, which served as an overall measure of the degree of bursting. A second metric quantified the degree of bursting using the coefficient of variation of the ISI (SD ISI/ mean ISI; [21]).

Where appropriate for each of the preceding analyses, statistical significance was determined using one-way, two-way and repeated measures ANOVAs with post-hoc Bonferroni-adjusted or LSD comparisons, paired and two-sample t-tests, Tukey’s HSD tests, chi-square tests and Pearson’s correlations. Significance was set at P < .05 for all comparisons. Errors and error bars are expressed as standard errors (SE) of the mean.

## Results

### General response characteristics (10s net sums)

**Figure 1:**
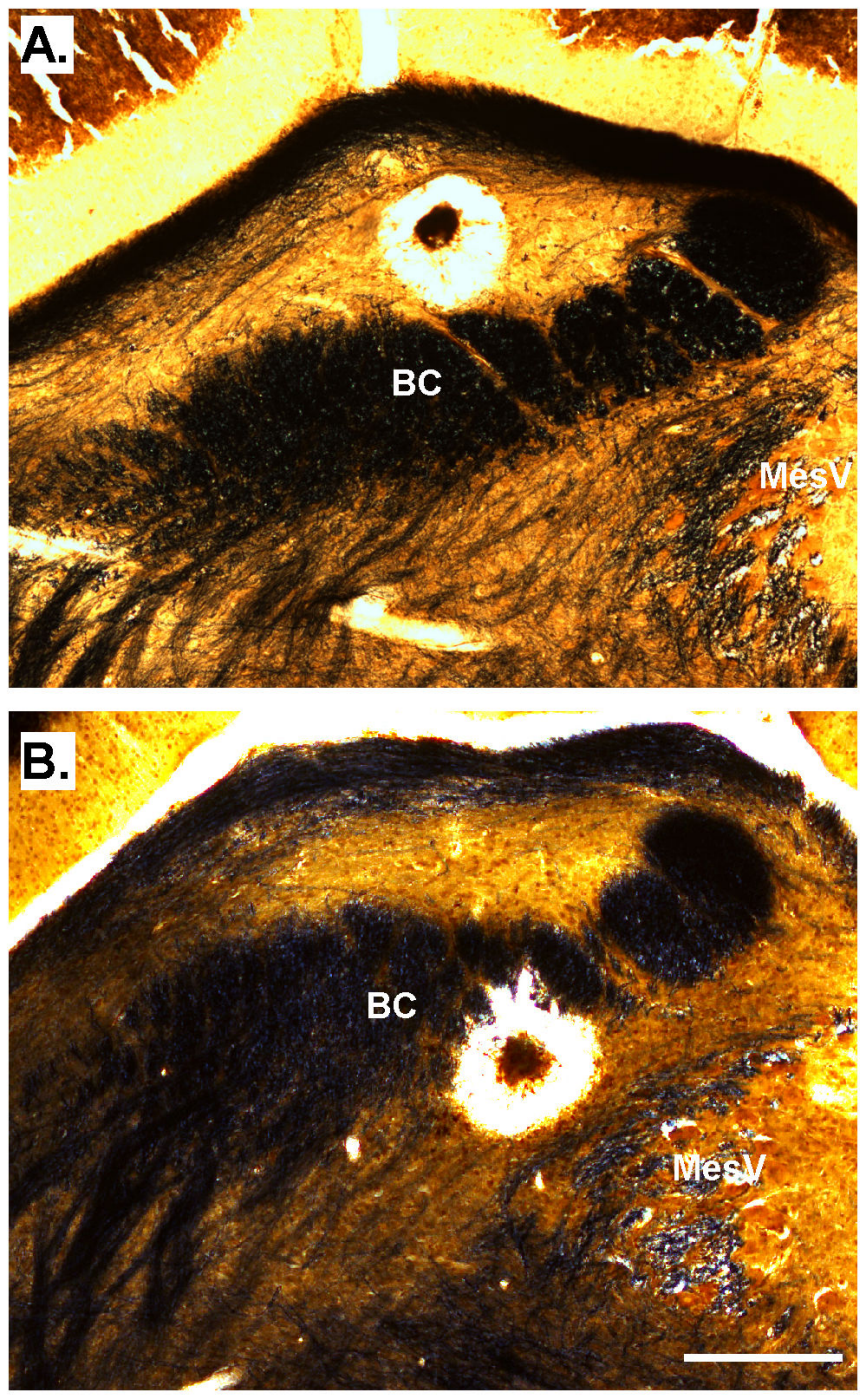
Representative recording sites. Photomicrographs of Weil-stained sections illustrating lesions made at the site of recordings. Myelinated fibers appear black; cellular areas are golden. A. Site of recording for the neuron presented in Figure 2. This neuron was recorded dorsal to the brachium conjunctivum in the ventral lateral subnucleus. B. Site of recording from the neuron presented in Figure 3; this cell was recorded ventral to the brachium conjunctivum in the medial subnucleus. Scale bar= 250µm. Abbreviations: BC = brachium conjunctivum, MesV=mesencephalic nucleus of the fifth nerve.

**Figure 2:**
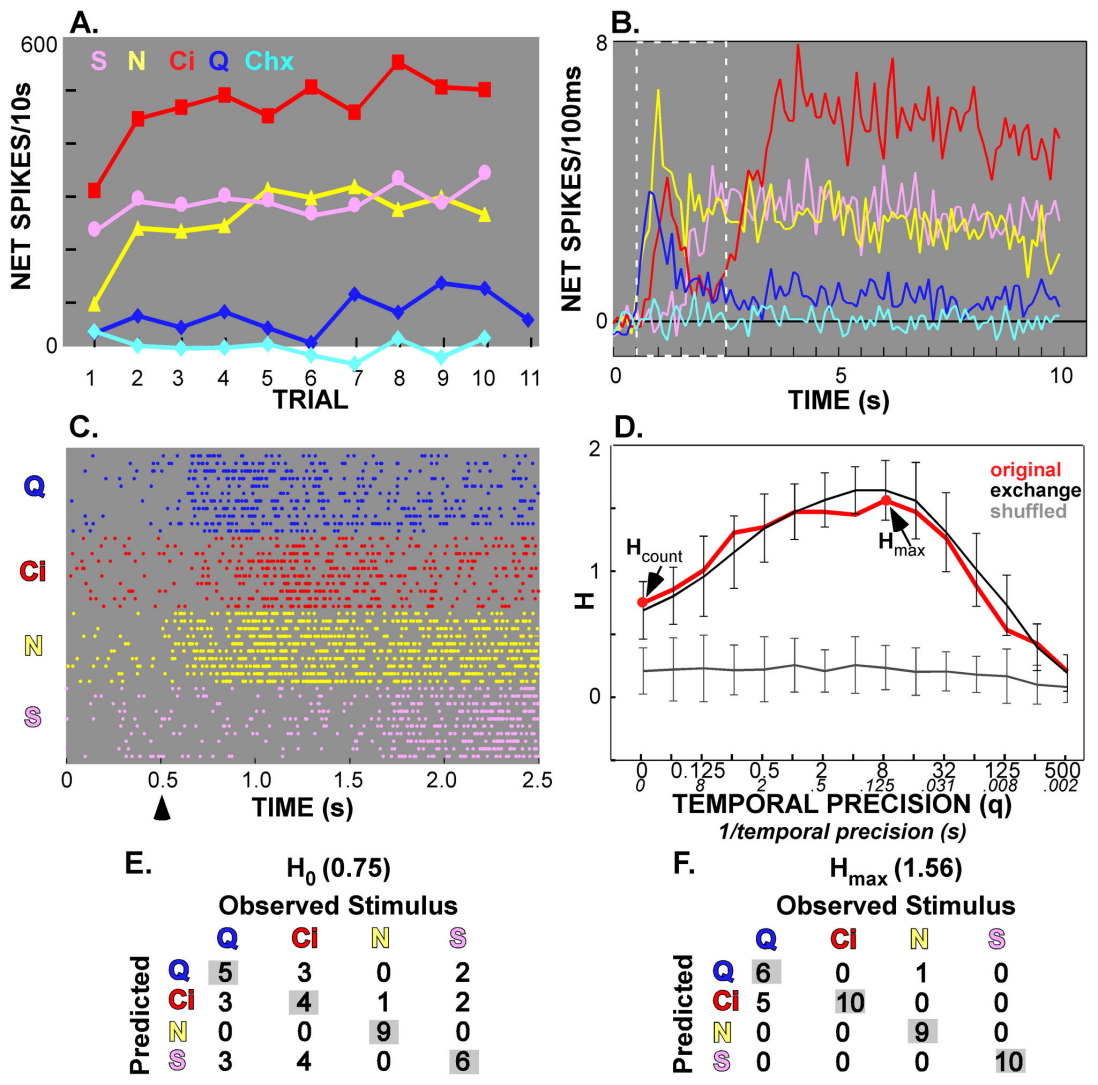
Example of a broadly-tuned AN neuron with a substantial contribution of timing to between-quality discrimination. (**A**) Over the 10s period, this cell responded most robustly to citric acid, but had substantial sidebands to sucrose and NaCl. Relative response magnitudes were stable across 10 trials. (**B**) Although acid was clearly the best stimulus over 10s, the mean response envelopes were very complex. Every stimulus, except cycloheximide (Chx) elicited a substantial response at some point in the initial 2s (outlined in white). **C**. A raster plot of repeated trials over the same 2s period. The arrow indicates the time of stimulus onset. The figure legend showing the color-coding for the stimuli in Panels A-C is as shown in Panel A. **D**. Metric analysis of the first 2s of the response (period outlined in white in Panel B and from 0.5-2.5s in Panel C) showed that taking temporal factors into account improved discrimination across the 4 taste qualities tested. Note that the amount of information available (*H*) from spike rate alone (*H*_count_, in red at *q*=0) was less than that due to spike rate plus temporal factors (*H*_max_, in red at *q*=8[1/*q*=0.125]). However, when the exchange control was performed, this value was not exceeded by the original data at *H*_max_, indicating that response envelope rather than precise spike timing was responsible for the increase in information (see Results section). This increase is also evident in Panels **E** and **F**, which show the confusion matrices for *H*_count_ and *H*_max_, respectively. Note that correct prediction of citric acid rose from 4 out of 10 trials to 10 out of 10 with inclusion of the response envelope. Likewise, sucrose prediction improved dramatically.

**Figure 3:**
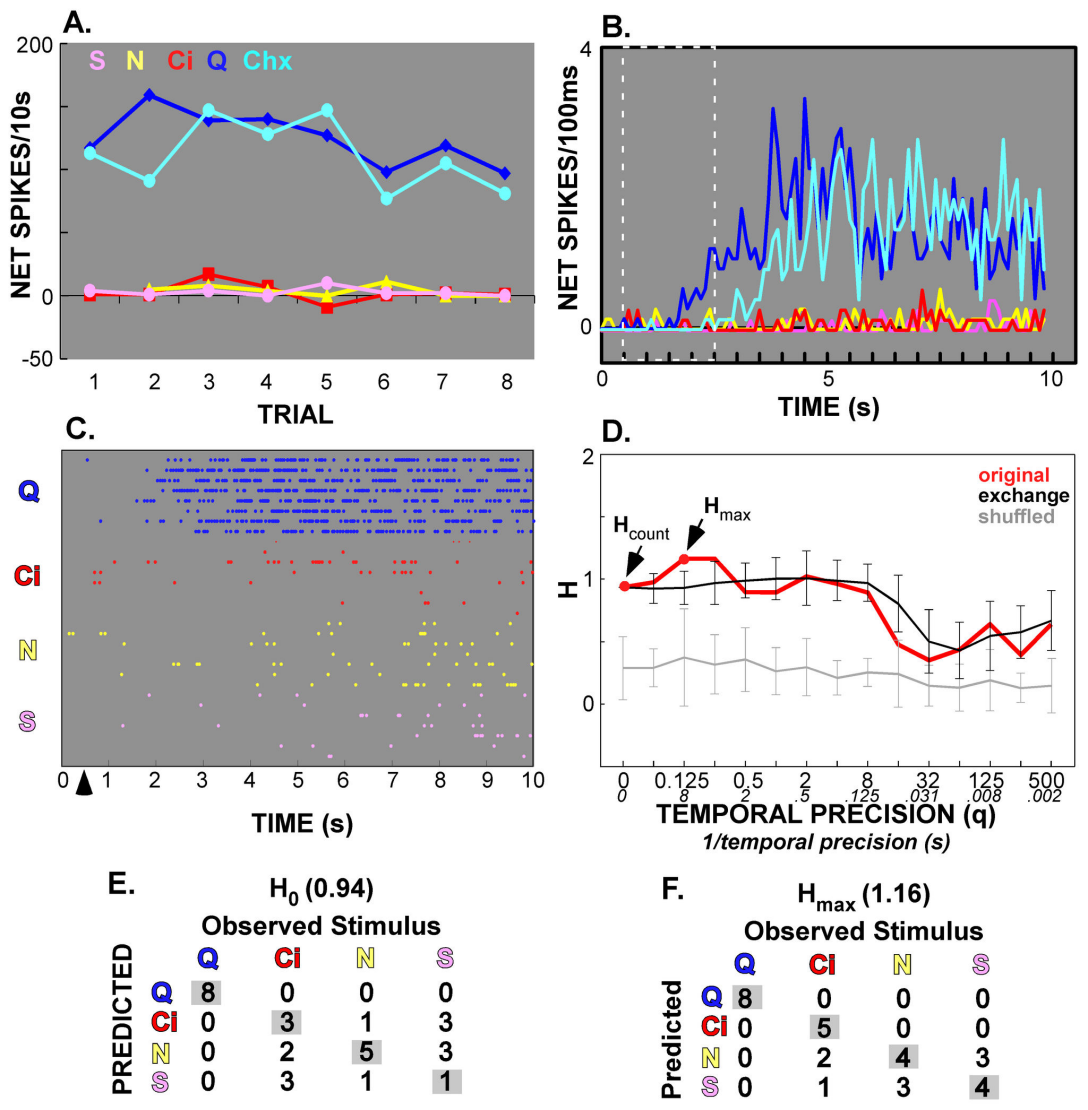
Example of a narrowly-tuned B-best neuron with a minimal contribution of timing to between-quality discrimination. **A** & **B**. Of the 4 standard taste qualities, this cell responded mainly to bitters (quinine and cycloheximide); this was true both across trials (**A**) and across the stimulation period (**B**). **C** & **D**. The entire 10s period was used in the raster plot (**C**) and metric space analysis (**D**), but the initial 2s period is outlined in white in panel B so that the slow onset of these responses can be contrasted with the example shown in Figure 1. The four-quality metric space analysis indicated a small improvement with the addition of temporal factors (i.e. *H*_max_ > *H*_count_), even though very few spikes were evident in the raster plot for non-bitter stimuli. **E** & **F**. The confusion matrices reveal that temporal factors increased the probability of correctly predicting both citric acid (increased from 3 to 5 correct predictions) and sucrose (improved from 1 to 4). The best stimulus, quinine, was not affected as it was discriminated perfectly by rate alone.

Twenty-nine neurons were tested with the 5 core stimuli. The mean spontaneous rate (± SE) over 10s was 70.6 ± 12.05 and mean net response rates summed over the 10s stimulation period were 49.7 + 14.30 for sucrose, 269.7 + 53.76 (NaCl), 165.6 + 29.71 (citric acid), 92.5 + 12.21 (quinine), and 36.3 + 14.01 for cycloheximide. Eighteen cells had receptive fields composed only of anterior mouth receptor subpopulations (AT or NID), 7 had mixed anterior and posterior inputs (FOL or SP), 1 was posterior only and 3 were unknown. Cluster analysis (Pearson’s r, average linkage) using either four or five stimuli separated neurons into classes that matched their best stimulus designation, except for one quinine-best cell that responded almost as well to NaCl and clustered with the N-best cells. For this reason, we named the clusters for the taste stimulus or quality that typically elicited the optimal response, according to the net 10s summed firing rate. Response characteristics of these groups are summarized in Table 1; average response profiles are discussed below. There were 4 S (sucrose)-best, 11 N (NaCl)-best, 8 AN (citric acid > NaCl), and 6 B-best (bitter; quinine and/or cycloheximide) neurons. Cluster status was systematically related to receptive field (Pearson’s chi square=22.8, p < .008) (Table 1). All AN and S-best cells received input only from the anterior oral cavity as did most N-best cells, while all B-best neurons received either mixed or posterior inputs. In addition, N-best cells were twice as likely to receive input from a single RF (the anterior tongue) while S- and B-best neurons usually received convergent input. Three of the 4 S-best cells received inputs from the anterior tongue and nasoincisor ducts, and 5/6 B-best neurons responded to the nasoincisor ducts along with the foliate papillae, soft palate, or both. Neurons of different best stimulus categories also varied significantly with regard to breadth of tuning (BOT), measured either using the entropy measure (BOT_H_) or the noise:signal ratio (BOT_N:S_). Similar to earlier studies, both measures revealed AN neurons to be the most broadly tuned, whereas B-best neurons were the most selective [11,13]. Figure 1 shows representative recording sites for two of the neurons recorded in the present study which are also used to illustrate temporal analyses in Figures 2 and 3. Both cells were located near the waist region of the PBN [22], one in the ventral lateral and one in the medial subdivision dorsal and ventral to the brachium conjuntivum, respectively. Recording sites were recovered for eight additional cases in nearby locations and the remaining neurons were encountered at coordinates that indicated that they were in the same vicinity.

**Table 1:** Response characteristics by neuron type^**1**^.

	**S-best**	**N-best**	**AN**	**B-best**	
**N**	4	11	8	6	
**Spontaneous rate**	16.1 (8.81)^a^	85.5 (21.11)^b^	119.0 (16.38)^b^	15.2 (7.11)^a^	ANOVA, P<.005
**Receptive**	AO only (n=4)	AO only (n=7)	AO only (n=7)	PO (n=1)	X^2^, P< .008
**Field (RF)**		Mixed (n=2)	Unknown (n=1)	Mixed (n=5)	
		Unknown (n=2)			
**BOT_H_**	0.549 (.132) ^ac^	0.677 (.047) ^ab^	0.790 (.098)^b^	0.477 (.085)^ac^	ANOVA, P<.02
**BOT_N:S_**	0.193 (0.078) ^ab^	0.404 (0.103)^b^	0.569 (0.048)^b^	-0.011 (0.272)^a^	ANOVA, P=.05
**CV_TR_ (overall)**	0.32 (0.07)	0.31 (0.04)	0.31 (0.040)	0.46 (0.09)	NS
**CV_TR_ (best stim)**	0.12 (0.03)^a^	0.10 (0.02)^a^	0.14 (0.01)^a^	0.24 (0.06)^b^	ANVOA P<.05
***H*_count_**	1.42 (0.04)	1.28 (0.1)	1.32 (0.12)	0.97 (0.05)	ANOVA, P=.07
***H*_max_**	1.55 (0.04)^a^	1.80 (0.05)^b^	1.81 (0.05)^b^	1.28 (0.09)^c^	ANOVA, P<.001
***H*_max_-*H*_count_**	0.135 (0.021)^a^	0.517 (0.06)^b^	0.49 (0.12)^b^	0.30 (0.07)^b^	ANOVA, P<.05
***H*_temp_**	8.7% (0.14)	29.4% (0.04)	27% (0.06)	22.5% (0.46)	ANOVA, P=.07
**Mean # of stimulus replications**	5.8 (1.03)	8.1 (0.68)	7.3 (.92)	7.3 (1.28)	ANOVA, P=.48

1 values represent mean (standard errors); superscripts with different letters indicate significant differences based on post-hoc LSD tests. Values represent 10s of activity and 4 taste qualities.

### Stability of 10s net responses

Our previous study of PBN neurons, which tested a subset of cells with 2-3 trials per stimulus, suggested that 10s net response profiles were stable over time [11]. The current study confirmed this conclusion with a larger sample and greater number of replications. Thus, although the average coefficient of variation across repeated trials (CV_TR_) was 0.34 + 0.028, 26 of 29 neurons responded optimally to the same stimulus quality across all trials (as shown in Figures 2A and 3A). Indeed, the mean CV_TR_ for the best stimulus was just 0.14 + 0.016, significantly lower than the CV_TR_ for the second-best stimulus, 0.33 + 0.06 (P<.005). This trend was consistent across best-stimulus groups (Table 1). Three of 29 (10.3%) neurons did change best stimulus over repeated trials but there were no obvious characteristics that differentiated these neurons from the others. Also, a repeated-measures ANOVA for firing rate during the pre-stimulus water application was not significant when the first and last trials were compared for each of the four taste qualities (P > .11), suggesting that the overall activity of the neurons remained stable over time.

### Response envelope

**Figure 4:**
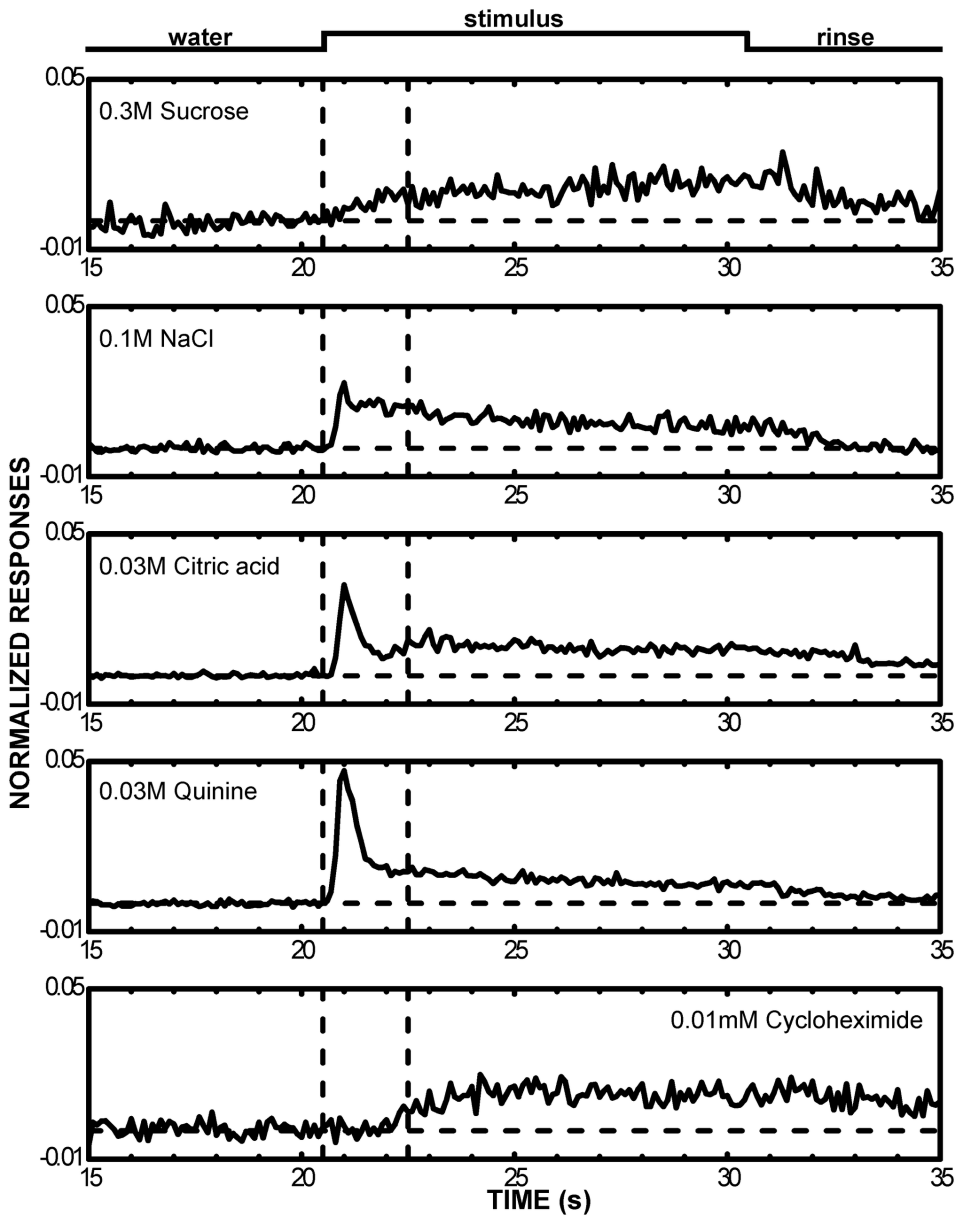
Mean time course varies with stimulus. Net spikes per 100ms were normalized to the 10s total and averaged across all cells for each of the 5 taste stimuli. Only cases where a significant response was elicited were included in the means (sucrose, n=15; NaCl, n=26; citric acid, n=27; quinine, n=21; cycloheximide, n=8). The onset and offset of the stimulus are shown at the top of the figure. The trial began at time 0, spontaneous rate was recorded for 10 s, then water baseline for 10 s. Water began flowing 5s prior to the period shown, the taste stimulus contacted the oral cavity at ~20.5 and the rinse started 10s later. The initial 2s period after stimulus onset is demarcated with dotted lines and corresponds with the interval analyzed by ANOVA. Note that electrolyte responses (NaCl, citric acid, quinine) had large initial phasic components, while the nonelectrolyte stimuli (sucrose and cycloheximide) did not.

Figure 4 depicts the response time course in 100ms bins for each stimulus averaged across all neurons responding significantly to a given tastant. Prior to averaging across cells, response rates in each bin were normalized to the 10s summed response to allow scrutiny of the shape of response envelopes while minimizing differences in magnitude. Time course varied with stimulus. Most notably, the three electrolytes: NaCl, citric acid, and quinine, elicited rapidly-peaking responses with pronounced phasic components whereas the non-electrolytes, sucrose and cycloheximide, elicited a slowly incrementing pattern. Moreover, among these stimuli, the proportion of the response contained in the initial phasic period was most pronounced for quinine and least pronounced for NaCl. Approximately 2s after stimulus onset, firing rates exhibited less fluctuation. A 2-way ANOVA for the first 2s of the response yielded a significant interaction between time and stimulus (P<.001) and subsequent 2-way ANOVAs for every stimulus pair except sucrose-cycloheximide showed significant interactions between stimulus and time (all P’s < 0.05), supporting the hypothesis of distinct, stimulus-dependent rate envelopes.

**Figure 5:**
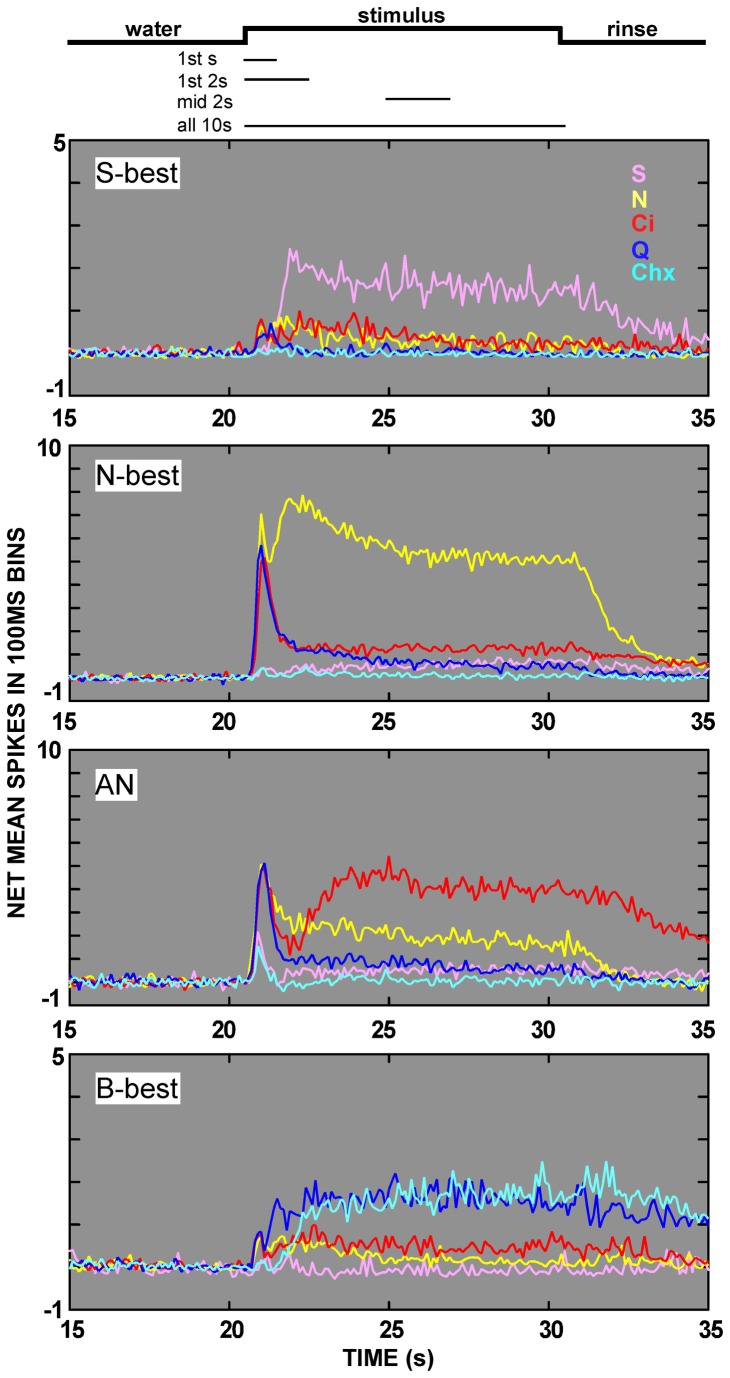
Mean time course is a function of neuron type as well as stimulus. Each panel shows net spikes per 100ms averaged across neurons of a given neuron type for each of the 5 taste stimuli. The stimulus time course is again shown at the top of the figure and the lines underneath indicate the times used for analysis. The N-best and AN neurons exhibited clear transient responses during this initial phase while B-best neurons did not, regardless of the electrolyte status of the stimulus. Furthermore, the phasic responses to the electrolyte stimuli (NaCl, citric acid and quinine) were very robust for N-best and AN neurons, with the best stimulus becoming clearer by the end of this initial 2s period. Note that the mean transient responses to sucrose and cycloheximide in the AN group are the result of 1 cell (neuron 47.1).

When mean time course was inspected separately for each neuron type, additional insights became apparent (Figure 5). All responses were included regardless of significance, and non-normalized net firing rates were used to allow examination of response magnitude as well as time course. Analyses of variance indicated a significant neuron group X time interaction for the initial 2s of the responses to sucrose (P<.05), NaCl (P<.001), citric acid (P<.001), and quinine (P<.001), as well as the first 3s of the response to cycloheximide (P=.003), indicating that the time course of each stimulus was related to neuron type.

Similar to the overall time course, NaCl, citric acid, and quinine elicited rapidly peaking responses with large phasic components in both N-best and AN neurons. However, the relative magnitude of the tonic response was dependent on both stimulus and neuron type. A larger tonic component characterized the salt response in N-best neurons and the acid response in AN cells but the tonic response to quinine was small in both groups. An ANOVA analyzing the tonic: phasic response ratio (i.e. the summed response for the middle second/first second) for NaCl, citric acid, and quinine in N-best and AN neurons indicated a significant main effect of stimulus (P < .001) and an interaction between stimulus and neuron type (P < .001). Post-hoc comparisons confirmed an effect of neuron cluster for each stimulus (all P’s < .001).

Distinctive characteristics were also apparent in the response envelopes of S- and B-best neurons. In sucrose-best cells, the latency to the peak of the response appeared shorter than for the population average. Moreover, it was interesting that small electrolyte responses were elicited in S-best neurons with an average response latency that was roughly 0.5s shorter than the response elicited by sucrose. Nevertheless, like the population average, sucrose elicited a less dramatic phasic response in S-best cells than electrolytes did in N- and AN neurons. In B-best neurons, the response envelope for cycloheximide was similar to the population average, likely because other neuron types generally failed to respond to this stimulus. In contrast, the quinine response was markedly different and lacked the dramatic phasic component observed for quinine responses in N- and AN cells. Instead, similar to cycloheximide, quinine elicited a slowly incrementing response in B-best cells, albeit one that peaked sooner.

### Ensemble coding over time

**Figure 6:**
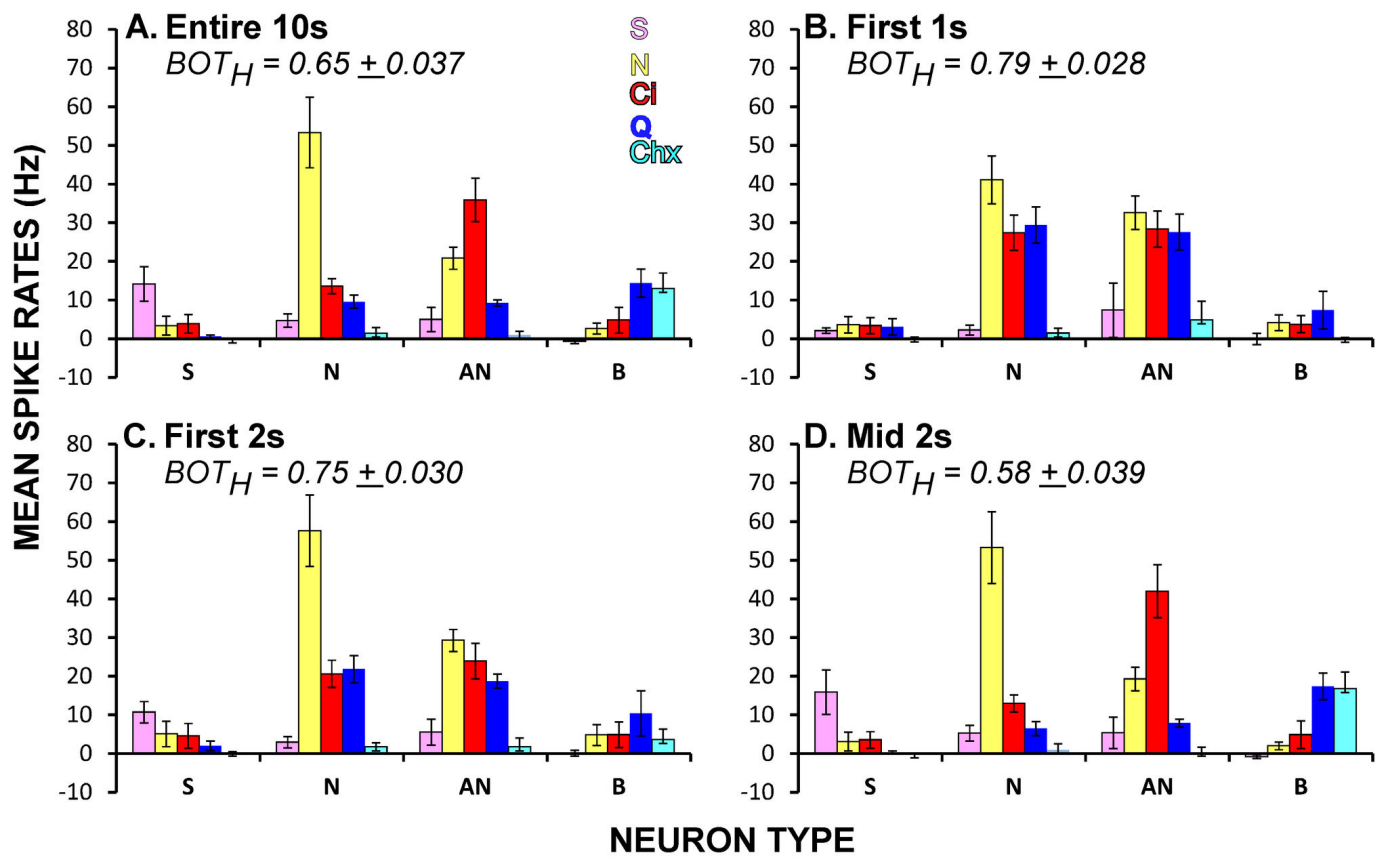
Response profiles change over time. **A**. Over the entire 10s taste period the best stimulus for each chemosensitive neuron type was readily identifiable. **B**. In contrast, when responses were limited to the first second, both N-best and AN neurons were broadly-tuned. **C**. When responses were summed over the first 2s, N-best neurons became more selective while the AN neurons were still quite broad. In addition, the profiles of S-best and B-best cells become more distinct. **D**. Profiles for all four types of neurons are quite distinctive using summed responses from the middle 2s and resemble profiles generated from the entire 10s. ANOVA showed a significant difference in the BOT_H_ for response profiles during the different time periods (P<.0005). BOT_H_ for all the time periods differed from one another (P’s < .005, Bonferroni adjusted), except for the first second versus the first 2s.

**Figure 7:**
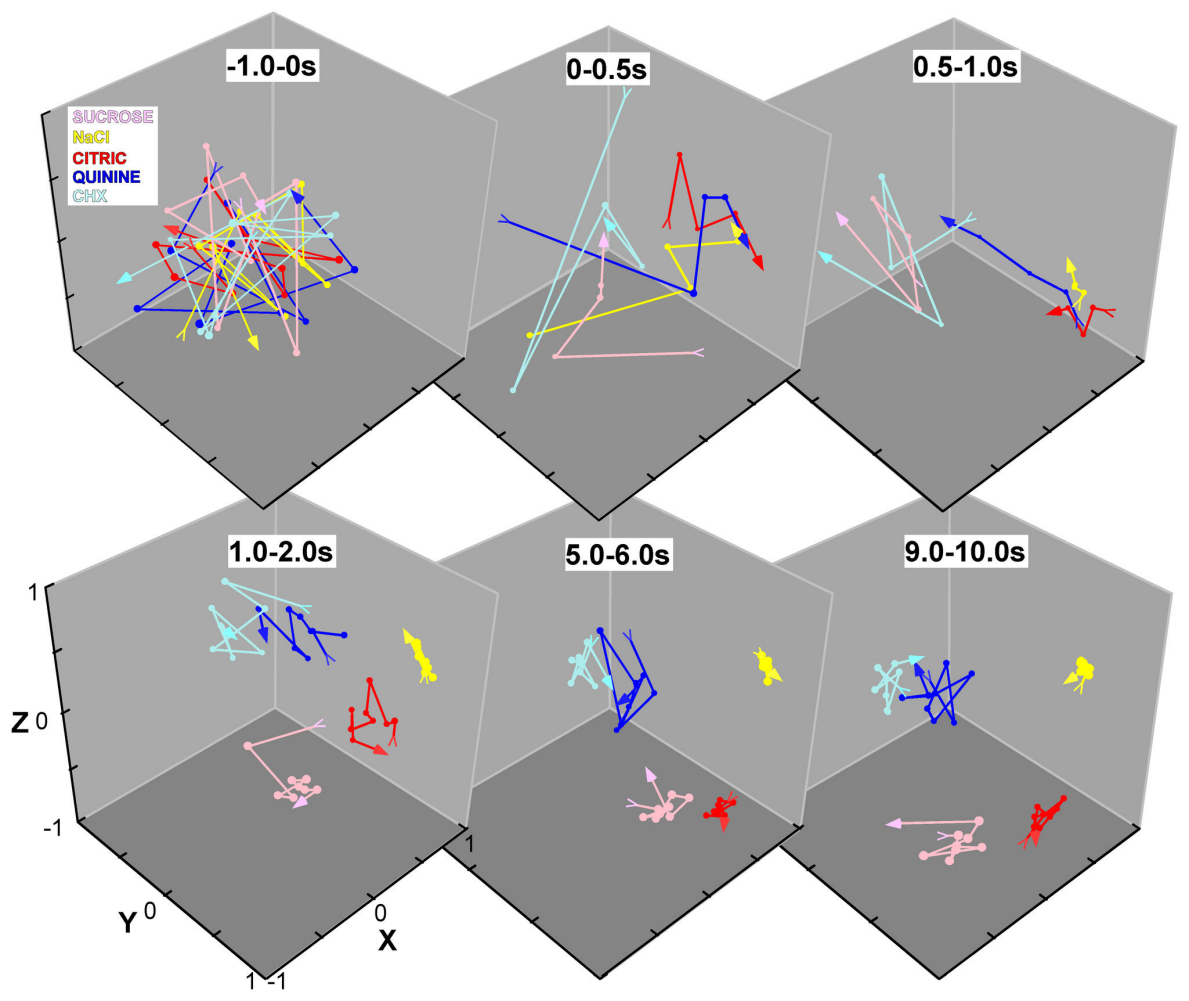
Evolution of the ensemble over time. Across-neuron correlations were calculated across stimuli and time (100ms bins). These are represented graphically using multidimensional scaling (MDS) for different time periods (0.5 or 1.0s in duration). The pre-stimulus activity and first 2s of the response are shown in their entirety; representative intervals are shown for the remainder of the taste period. The initial and final time points for each stimulus in a given epoch are marked with a tail and arrow, respectively. The 1^st^ graph illustrates the second immediately prior to taste onset (i.e. the final second of pre-stimulus water). No correlated activity was obvious at this time (top left graph). During the first 500 ms (0-0.5s) electrolytes segregated from non-electrolytes and then began to move away from each other, a trend that continued over the next 500 ms (0.5-1s). In the initial part of the next epoch (1.0-2.0s, bottom left), all four qualities were clearly segregated. Over the next few seconds (not shown), these groups became slightly more cohesive and by seconds 5-6 (bottom middle) were not different from those observed during the last second of stimulation (bottom right). The three axes depict arbitrary units of distance.

The dynamic time course of PBN taste responses impacted the nature of the chemosensitive response profiles characterized at different time points. Figure 6 shows mean response profiles for the 10s period used for classifying neuron groups and the profiles for these same groups during the early and middle portions of the response. Response profiles were broader initially, particularly in the first second of the response, suggesting time-dependent shifts in the ensemble code. To examine how coding changed over time, we calculated across-neuron and time correlations for the spontaneous period and each subsequent 100ms bin and used multidimensional scaling to visualize the relationships. Figure 7 shows the evolution of the response space for selected time points. Before stimulation, across-neuron correlations between successive 100ms bins and stimuli were highly variable, with no systematic relationships. During the initial 500ms after stimulus contact, the three electrolytes became closely aligned, presumably reflecting the large phasic responses elicited by NaCl, citric acid, and quinine in both N- and AN neurons. Subsequently, however, quinine rapidly separated from salt and acid and moved toward the other bitter stimulus, cycloheximide, and salt and acid began to separate from one another. Just after the first second had elapsed, stimuli representing all four qualities formed clear clusters in the MDS space. After this initial period, changes were subtle although the bitter cluster continued to constrict until about the fourth second, apparently reflecting the slowly-rising responses evident in B-best neurons. Nearly identical results were obtained when the same data were analyzed using a principal components analysis (not shown).

### Metric space analysis: 4 distinct qualities

**Figure 8:**
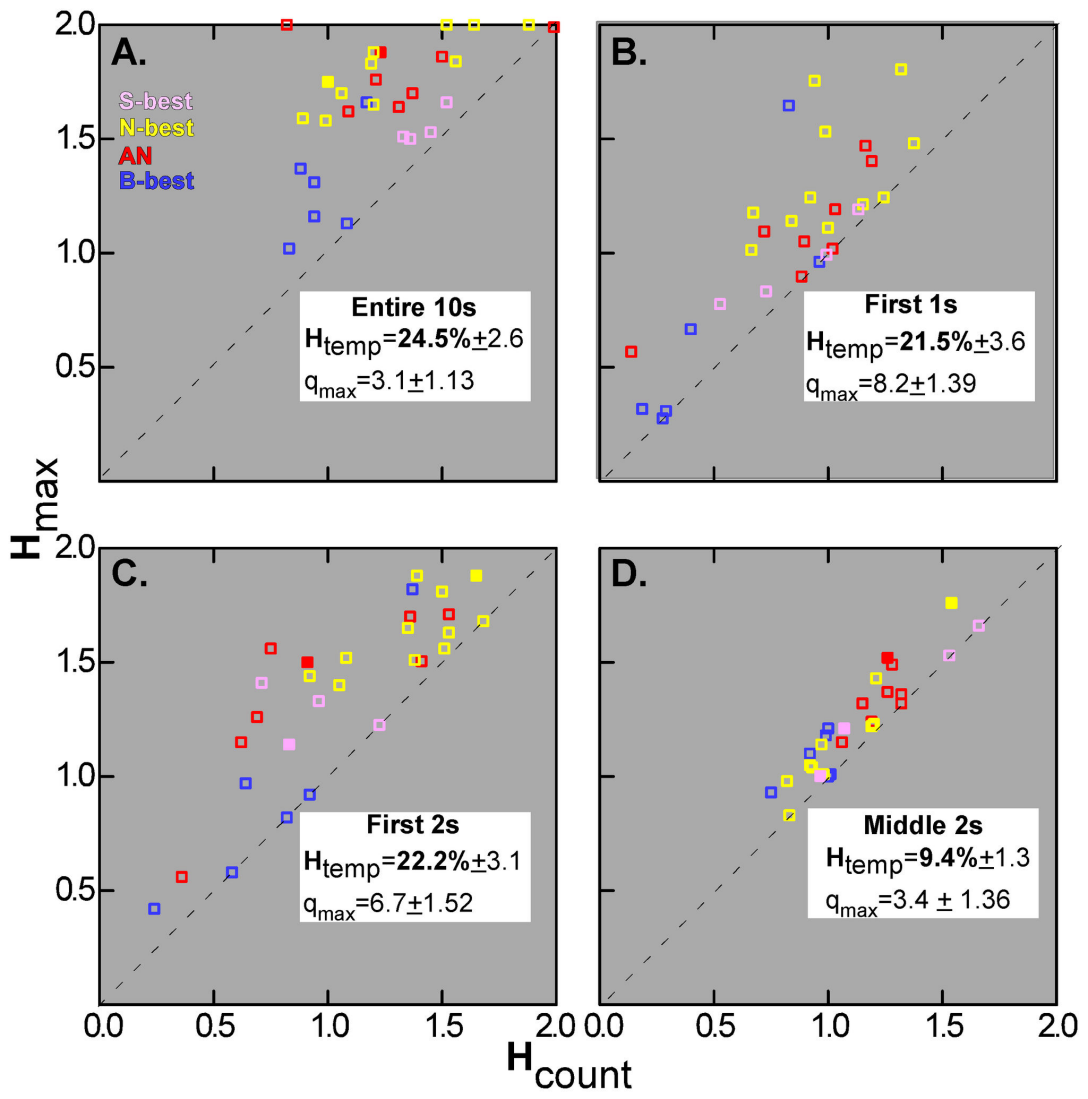
Metric space analysis of between-quality discrimination: *H*_max_ vs. *H*_count_ by time period and stimulus. Maximum information (*H*_max_) is represented on the y-axis and the amount of information due to spike rate alone (*H*_count_) is on the x-axis for each individual neuron. Neurons are further classified according to best stimulus (denoted by color) and by whether the exchange analysis was significant, indicative of a contribution of precise spike timing (closed symbols). Mean *H*_temp_ and *q*_max_ values are also given for each analysis; *H*_temp_ is markedly lower for the middle 2s period. Overall, *H*_max_ exceeded *H*_count_ and precise spike timing was less prevalent than rate envelope. Even when timing was taken into account B (bitter)-best, in blue, and S (sucrose)-best cells, in pink, provided less information than neurons optimally responsive to salts and acids (yellow and red, respectively).

The preceding findings suggest that temporal aspects of firing impact the ensemble coding of taste quality in PBN. However, using metric space analysis and information theory, recent studies have also provided evidence that temporal features of firing are likewise important for the information conveyed by single gustatory neurons in the brainstem [8,9]. We therefore used similar analyses to test this proposition for the current PBN data. Because previous studies have concentrated on the initial 2s of the response and because the results reported above suggested a shift in coding strategy with time, we analyzed the initial 1s and 2s periods, along with the middle 2s and the entire 10s response. Figure 8 depicts scatterplots of the information content contributed by spike count alone (*H*_count_) versus the maximum amount available with the inclusion of temporal factors (*H*_max_). Regardless of time window, most points fell above the diagonal and paired t-tests comparing *H*_count_ to *H*_max_ were significant for each period (all P’s < .001). Nevertheless, ratio scores [*H*_temp_ = (*H*_max_-*H*_count_)/*H*_max_] quantifying the degree to which temporal factors played a role, suggested variation in the contribution over time. The mean *H*_temp_ was 24.5% for the entire 10s period, 21.5% for the first s, 22.2% for the first 2 s, and 9.4% for the middle 2s (ANOVA, P < .001). Post-hoc comparisons revealed that significantly less information was provided by temporal factors during the middle 2s (seconds 5 to 7) than during the other time periods (P’s < .01, Bonferonni-adjusted). Median values of *q* at *H*_max_ (*q*_max_) tended to be larger for the initial time periods (ANOVA, P = .05). To distinguish between contributions of rate envelope and precise spike timing, exchange resampled data sets were compared to the original data. As indicated by the filled symbols in Figure 8, *H*_max_ exceeded the exchange value by 2 standard deviations in 2 neurons for the 10s period (7.0%), none for the 1s period, 3 for the 2s period at response onset (10.3%), and 2 for the middle 2s (7.0%). This suggests that precise spike timing contributed to the increase in mutual information in a minority of cases and that rate envelope was the major factor conveying the additional information.

#### Detailed analysis of the 10s period

Figure 8 indicates a considerable range in the degree to which *H*_max_ exceeded *H*_count_. Because the increase in *H*_max_ was nominally greater over the entire 10s period, we focused on this time frame to analyze the relationships between metric space measures and other characteristics of PBN neurons (Table 1). There were differences in the maximal amount of information (*H*_max_) conveyed by different chemosensitive groups. B-best neurons conveyed the least information, and N-best and AN neurons the most. Although similar trends were apparent for information conveyed by spike count (*H*_count_), these differences were not significant, suggesting that temporal factors were necessary to fully exploit potential information in the broader N- and AN neurons. Indeed, the absolute difference by which *H*_max_ exceeded *H*_count_ was significantly larger for N- and AN cells than for S-best neurons. A similar but non-significant trend was apparent for a related measure, the proportional increase in information (*H*_temp_). Thus, the two most broadly-tuned best stimulus classes showed the largest increases in *H*_max_. However, across all neurons, neither measure of breadth of tuning (i.e., BOT_H_ and BOT_N:S_) was significantly correlated with *H*_temp_ or *H*_max_-*H*_count_ (P’s > .05). Likewise, variability in spike rate across trials, as quantified by the CV_TR_, was not correlated with the degree to which information content improved with temporal factors (P>.05). The mean *q* value at *H*_max_ was 3.1 + 1.14, corresponding to a temporal precision of 1/*q* = 323ms. Not only did this value range widely across the population (0.063-32), but its range was often quite broad for individual cells, i.e., *H* could be at its maximal value across several adjacent *q*’ s (see Figures 2 and 3).

Figures 2 and 3 (Panels C-F) present representative examples of the metric space analysis. The example in Figure 2 shows a neuron (labeled “Neuron 71.2”) that was classified as an AN cell and was one of the most broadly-tuned in the present sample. Responses exhibited complex patterns of firing over time, particularly for acid (Figure 2B, C). The proportion of the maximal information contributed by temporal factors was 0.31 for the entire 10s period, 0.21 for the first s, 0.52 for the first 2s but only 0.08 for the middle 2s. Panel D presents the metric space analysis for the first 2s, the period when time made the largest contribution. Using spike count alone, the information conveyed (*H*_count_) was just 0.75 out of a possible 2.0, and NaCl was the only stimulus predicted with any accuracy (Panel E). Interestingly, citric acid, the most effective tastant over the 10s period, was often misclassified during the early response, presumably because all three electrolytes exhibited robust phasic peaks and because acid did not become the clear best stimulus until later in the stimulation period. Temporal factors improved information content to 1.56 at a temporal precision (1/*q*) of 125ms, and yielded good prediction for all stimuli except quinine (Panel F). The example shown in Figure 3 (labeled “Neuron 55.2”) was a much more narrowly tuned B-best neuron that responded optimally to quinine (and cycloheximide), with a long-latency, slowly-peaking response (Figure 3B, C). The other stimuli elicited tiny, less consistent responses. The proportion of information contributed by temporal factors (*H*_temp_) for the entire 10s period was 0.19 and was negligible for the other time windows analyzed (*H*_temp_ < 0.06). Over the 10s period, *H*_count_ was 0.94 and spike rate correctly predicted the best stimulus, quinine, on 10 of 11 trials (Figure 3D). Not surprisingly, however, the other stimuli were poorly discriminated. With temporal factors included, information content rose slightly (*H*_max_ = 1.02, 1/*q* = 8s), with sideband stimuli correctly identified somewhat more frequently.

### Metric Space Analysis: Two stimuli of the same quality

**Figure 9:**
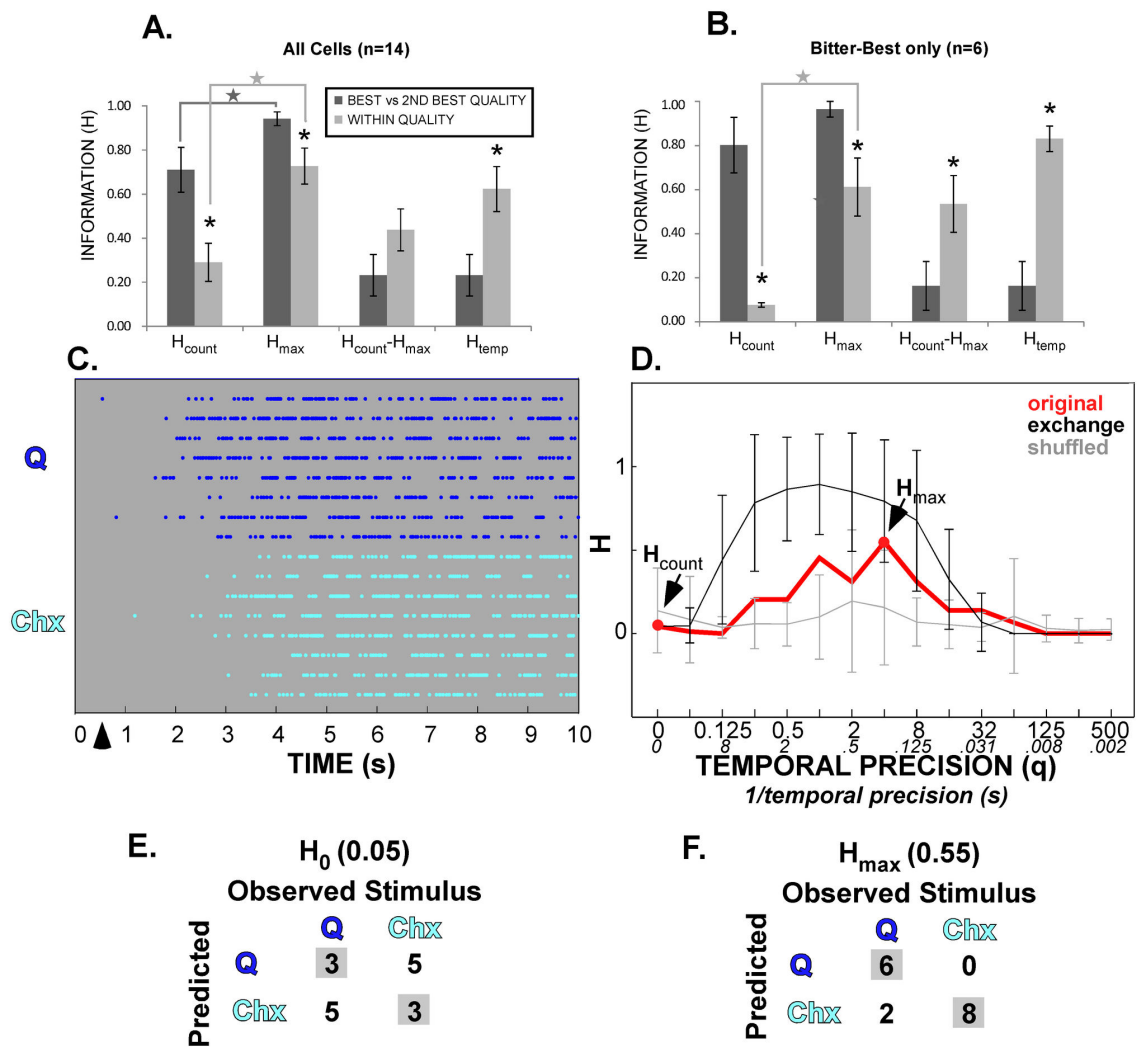
Metric space analysis of within-quality discriminations. **A** & **B**. Bar graphs compare information metrics for two stimuli of the same quality (within) vs. the two most effective stimuli of different taste qualities (best vs 2nd-best) for all neurons (**A**) and bitter-best neurons alone (**B**). Note that with only two stimuli the maximal *H* value possible is “1”. The within-quality discrimination yielded lower values for mutual information than the between-quality discrimination regardless of whether spike rate alone was considered (*H*_count_) or whether time was taken into account (*H*_max_). Nevertheless, temporal factors increased information content for both types of discrimination. In fact, the proportion of information contributed by timing (*H*_temp_) was greater for the within-quality discrimination. These differences were exacerbated for the bitter-best cells, with the absolute difference between *H*_max_ and *H*_count_ becoming significant for this population. **C**. Representative raster plot for responses to quinine and cycloheximide for the B-best neuron shown in Figure 2. **D**. Metric space analysis for the within-quality discrimination for this cell. **E** & **F**. Associated confusion matrices for *H*_count_ and *H*_max_ for this analysis. For this neuron, time contributes substantially to discriminating between the two bitter stimuli, whereas it had little impact on between-quality discrimination (Figure 2). The raster plot indicates overall similarities (long latencies, lack of a phasic response, bursting) between the quinine and cycloheximide responses; but the cycloheximide responses consistently begin later. Interestingly, the exchange control values significantly exceed the real data (**D**), strongly implying that the improvement with time is attributable to response envelope instead of precise spike timing. Thus, it seems plausible that the longer-latency associated with cycloheximide is responsible for ability of the cell to discriminate between the two stimuli.

Fourteen cells from our dataset were also queried as to whether time could contribute to within-quality discrimination. In some cases, these neurons were stimulated with an additional tastant representing the neuron’s putative best stimulus category. This extra stimulus was worked into the trials such that, in the majority of cases, the same number of replications for the extra stimulus was completed as for the standard five stimuli. Five AN neurons were probed with the citric acid standard and either 10mM HCl (n=4) or 0.1 M NH_4_Cl (n=1), and two S-best cells were stimulated with 20 mM Na-saccharin in addition to sucrose. As all cells were regularly stimulated with 2 bitter stimuli (quinine and cycloheximide), an additional stimulus was not required for the 6 B-best neurons. A lone N-best neuron strongly responsive to bitter stimuli was also tested for within-bitter discrimination. As in the between-quality data set, each stimulus was tested between 3 and 12 times (mean + s.e.m. = 7.2 + 0.7). We performed metric space analyses of the 10s period to determine if temporal factors contributed to discrimination between two stimuli with a shared taste quality. Indeed, similar to the four-quality discrimination, *H*_max_ significantly exceeded *H*_count_ for the within-quality discrimination across cells (P=.009, Figure 9A). However, *H*_max_ for the original data significantly exceeded that of the exchange resampled data set in only 1 of 14 cases (7.1%), suggesting that again, that rate envelope was the major temporal factor. To assess the influence of temporal factors in distinguishing stimuli of the same versus different qualities, we compared metric space measures for the within-quality discrimination to those for discrimination between the best- and the second-best stimulus for these same 14 cells. As for the within-quality discrimination, *H*_max_ significantly exceeded *H*_count_ for the best- versus second-best discrimination. Not surprisingly, however, since concentrations of the two representative stimuli for a given quality were chosen to be equally effective, the information contributed by spike count alone was significantly greater for between- than within-quality discriminations (P=.013). In addition, the average *H*_max_ for the between-quality discrimination was nearly perfect and significantly greater than *H*_max_ for within-quality discrimination (P=.022). In contrast, the *proportion* of information contributed by temporal factors was nearly 3-fold higher for within-quality pairs (63% vs 23%, P=.001). Next, the analysis was restricted to B-best neurons and the cycloheximide – quinine discrimination. The trends reported above were magnified for these narrowly-tuned cells (Figure 9B). The average *H*_count_ for B-best neurons was very high for between-quality discriminations but very low for the quinine/cycloheximide pair. Strikingly, including temporal factors increased the ability of B-best cells to distinguish quinine from cycloheximide by nearly 8-fold, and both *H*_count_-*H*_max_ and *H*_temp_ were significantly higher for the within-quality discrimination (p=.029 and P=.001, respectively). Figure 9C shows the raster plots and metric space analysis for the within-bitter discrimination of the same neuron that was presented in Figure 3 as an example of between-quality discrimination. The neuron has very similar 10s spike counts for quinine and cycloheximide, and discrimination between these stimuli was nearly random based on firing rate. In marked contrast, when temporal factors were included, quinine and cycloheximide were correctly identified 88% of the time.

### Bursting patterns of firing

**Figure 10:**
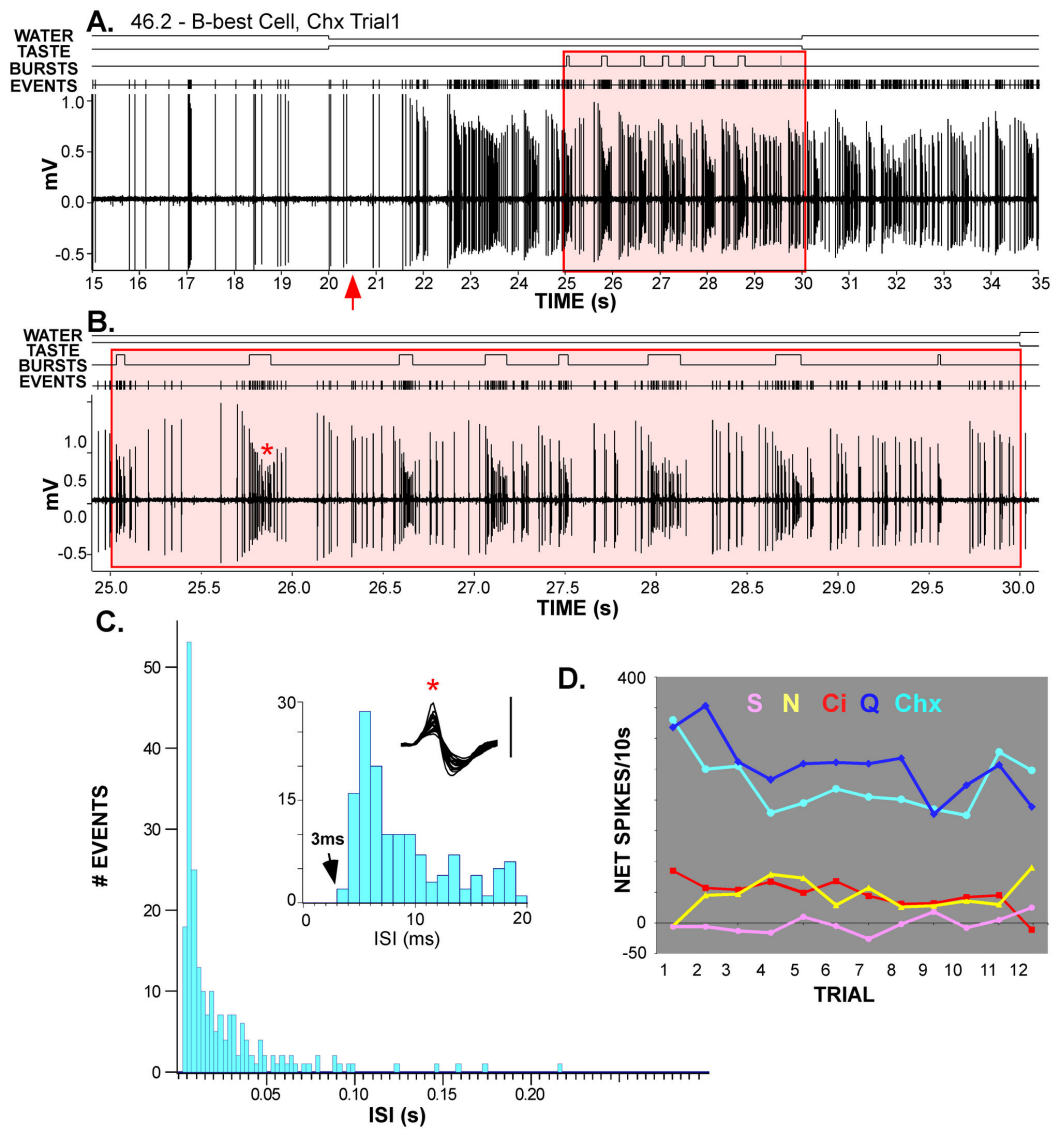
Example of a B-best neuron with a bursting firing pattern. **A** & **B**. Raw record of a bitter-best neuron responding to 0.01 mM cycloheximide. Panel **A** extends from the last 5s of water through the 10s taste period and 5s into the rinse phase. The red arrow signals the tastant reaching the oral cavity and the highlighted region indicates the period used for the burst analysis (i.e. the last 5s of the taste period). Action potentials also appear as events in the channel above the raw record, with bursts identified in a separate channel. Panel **B** shows only the last 5s of the taste period (area outlined in red in Panel **A**). **C**. An ISI histogram of this 5s period using 2.5 ms bins showed a modal ISI of 10 ms and a skewed distribution, indicative of a bursting pattern. The inset depicts the shortest ISI’s at a finer grain (1ms), and the waveform of the action potential (taken from the burst marked with a red asterisk in Panel B). The shortest recorded ISI was >3 ms (i.e., spikes did not occur during the refractory period). Moreover, the waveform was consistent despite marked attenuation in spike amplitude during bursts, indicating that the windowed spikes came from a single cell. (**D**) This neuron responded robustly to quinine (dark blue) and cycloheximide (light blue) across trials and minimally, albeit significantly, to citric acid (red) and NaCl (yellow). It did not respond to sucrose (pink). Scale bar for the inset in Panel C = 100µV.

**Figure 11:**
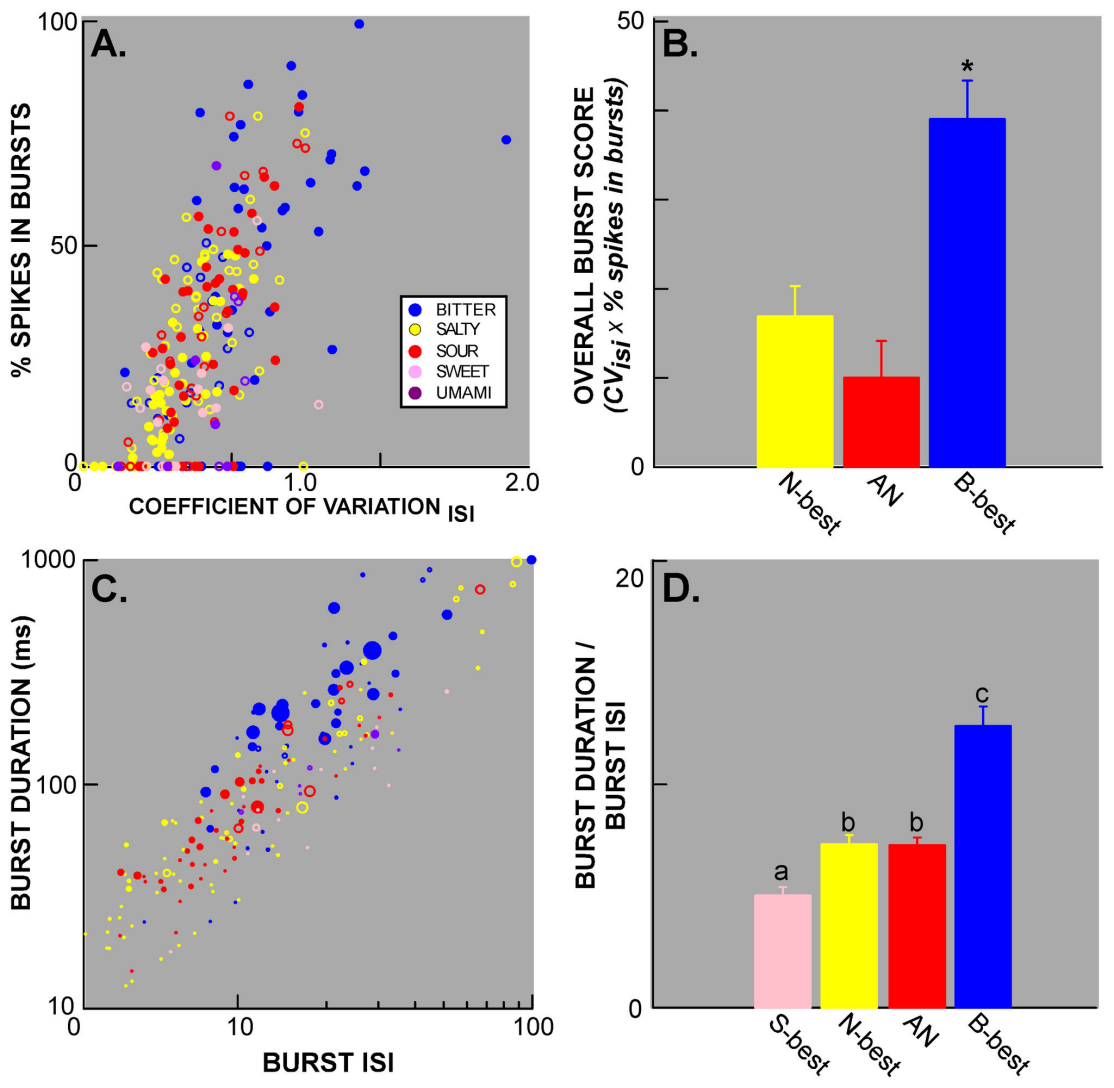
Burst measures according to stimulus and neuron type. **A**. Scatterplot of the two burst measures, % of spikes in bursts and coefficient of variation of the ISI, for significant individual responses from neurons recorded in this and a previous experiment [11]. Symbols are color-coded according to taste quality and filled symbols indicate that the stimulus was optimal for driving a given neuron. A subset of responses to bitter stimuli (blue) overlapped responses to other tastants while other bitter responses exceeded these values for one or both measures. **B**. Mean overall burst scores (CV_ISI_ x % of spikes in bursts) for responses to bitter stimuli arising from N-best, B-best and AN cells. Responses to bitter stimuli are significantly burstier in B-best neurons. **C**. Burst duration plotted against burst ISI and represented on a log scale for clarity. Symbols are color-coded for taste quality as in A. The size of the symbol is scaled according to the overall burst score. For a given ISI within bursts, bursts are longer for bitter-evoked responses. **D**. Mean + SEM normalized burst duration (burst duration/burst ISI) for all significant responses in a given neuron group. B-best neurons exhibited responses with longer bursts than the other three types of cells and S-best neurons were associated with shorter bursts.

During data collection for this and our previous investigations [11,13], we noticed another interesting temporal feature of brainstem gustatory responses; namely that bitter tastants often seemed to elicit a distinctive bursting pattern of firing (Figure 10). However, this phenomenon had not been formally explored. As described in the *Methods*, because multiple trials were not essential, these data came not only from the neurons analyzed above, but also from cells rejected due to low numbers of stimulus trials and from a previous PBN study [11]. We simplified the classification scheme from the earlier study (which defined 6 chemosensitive groups) and divided all 122 neurons into the 4 clusters described above (S-best, n=11; N-best, n=41, AN, n=41, and B-best, n=29). Figure 11A is a scatterplot of individual responses plotted according to degree of bursting measured by the coefficient of variation of the interspike interval (CV_ISI_) and percentage of spikes occurring in bursts (% spikes in bursts), as defined by the Poisson surprise criterion. Responses to bitter (cycloheximide and quinine) and other stimuli overlap, but the highest scores for both measures were associated with bitter responses. Analyses of variance for both these measures, an overall burst score combining them (OBS = CV_ISI_ X % spikes in bursts), and the average S (surprise) value for identified bursts confirmed this impression (Table 2). Similarly, B-best neurons had significantly higher scores on burst criteria than other cell types (Table 2). Next, bursting was specifically compared for bitter responses across the responsive neuron types, B-best (n=40), AN (n=10), and N-best (n=8) neurons (Figure 11b). An ANOVA indicated higher overall burst scores in B-best cells (ANOVA, P < .002; post-hoc LSD comparisons, B-best> AN and N-best, P’s<.02). Because nearly all responses to bitter stimuli in the AN and N-best groups were elicited by quinine, whereas both quinine and cycloheximide were efficacious in B-best neurons, a separate analysis was performed for quinine responses to make certain that this difference did not represent a distinction between quinine and cycloheximide. Even when restricted to quinine responses, B-best neurons exhibited higher burst scores (ANOVA, P<.00015; post-hoc LSD comparisons, B-best> AN and N-best, P’s<.0005). Finally, we conducted a within-neuron comparison for responses to bitter and sour stimuli in the subset of B-best (n=9) and AN neurons (n=10) that responded significantly to both stimuli. There was a main effect of neuron type (P<.03) but no effect of stimulus (P>0.1) and the interaction just missed significance (P=.06), suggesting that the propensity to burst is more strongly influenced by neuron type than stimulus quality, although both factors may contribute. Bursting responses to bitter stimuli in B-best cells were not only more prevalent, but also were distinctive. Figure 11C plots burst duration by burst ISI for individual responses. Across the population, both burst duration and burst duration normalized by burst ISI were significantly longer for responses to bitter stimuli and for responses from B-best neurons (Table 2; Figure 11D). Interestingly, although bitter responses were characterized by a higher degree of bursting, as defined by the percent of spikes occurring in bursts and the CV_isi_, responses to sour stimuli were characterized by a larger number of short-duration bursts (Table 2).

**Table 2:** Anova results for burst analysis^**1**^.

**MEASURE**	**TASTE QUALITY**			**NEURON TYPE**	
	**ANOVA, P**	**Post-hoc**		**ANOVA, P**	**Post-hoc**
		**comparisons^2^**			**comparisons^2^**
**Overall burst score (n=231)**	<0.000005	b>sa,so,sw,u		<0.000005	B>AN,N,S & A>N,S
**% spikes in bursts (n=231)**	0.00008	b>sa,so,sw,u & so>sw		<0.000005	B>AN,N,S & A>N,S
**CV_ISI_**	<0.000005	b>sa,so,sw		<0.000005	B>AN,N,S & AN>N
**Mean S ("surprise") value (=189)**	<0.000005	b>sa,so,sw & so>sw		<0.000005	B>AN,N,S
**Burst duration (n=189)**	0.0001	b>sa,so,sw		<0.000005	B>N,S,& AN>N
**Burst duration/burst ISI (n=189)**	<0.000005	b>sa,so,sw,u		<0.000005	B>AN,N,S & N, AN>S
**Number of bursts (n=231)**	0.00011	so>b,sa,sw,u& sasw		0.00069	AN>B,S & N>S
**Burst ISI (n=189)**	0.06813			0.00016	N>AN,B,S

^1^Abbreviations: Taste qualities: b=bitter, sa=salty, so=sour, sw=sweet, u=umami; Neuron types: AN=AN, B= B-best, N=N-best, and S=S-best. ^2^Fishers LSD; significant comparisons (all P’s < .05).

## Discussion

### Evolution of Ensemble Coding over Time

The present data support the growing consensus that temporal features of firing impact the neural representation of gustatory quality and further specify the nature of this influence. Analysis of the ensemble representation of quality at a fine temporal resolution demonstrated that the most dramatic changes occurred over approximately the first second of elicited activity. Interestingly, the first few hundred milliseconds after stimulus onset functioned mainly to separate electrolytes from non-electrolytes, prior to segregating individual taste qualities from one another. Not only did “salty” NaCl, “sour” citric acid and “bitter” quinine initially intermingle in the multidimensional representation, but quinine and the non-electrolyte bitter tastant, cycloheximide, were entirely separated from each other. Subsequently, quinine and cycloheximide became more closely aligned and NaCl, citric acid, and sucrose segregated from the bitter stimuli and each other. The fine-grained analysis of the response envelopes for different neuron types provides strong clues for the underlying basis of this evolution in the ensemble response. N-best and AN neurons achieved their peak firing rates quickly, but in the early part of the response responded robustly to each electrolyte and very weakly to the non-electrolytes, whereas B-best and S-best neurons were minimally active despite small electrolyte-driven responses in S-best cells. Changes then took place simultaneous with the segregation of the four qualities such that N-best and AN neurons responded less vigorously but more selectively to NaCl or citric acid depending on their optimal stimulus, while S- and B-best neurons accelerated firing in response to sucrose and bitter stimuli, respectively. Overall, a distinctive firing rate-based ensemble representation of quality emerged concurrently with more chemospecific tuning.

The slower responses of S- and B-best neurons are probably due in part to events at the receptor level, since taste receptors for sweet and bitter stimuli are GPCRs, while salts and acids use ion channels (see [23] for review). These comparatively sluggish responses are probably also a consequence of the more posterior location of the receptive fields for these cells. Similar, predominantly tonic responses have been observed in the mouse glossopharyngeal nerve (GL), which innervates the back of the tongue, indicative of a peripheral component [24] and GL response latencies to sweet and bitter stimuli have been reported to be as long as 10s [25]. All B-best neurons in the present sample had receptive fields that included the GL-innervated foliate papillae and/or posteriorly-located soft palate, but this was seldom true for N-best or AN neurons. N-best and AN cells exhibited a more rapid initial peak with phasic/tonic response envelopes and profiles resembling those described in the geniculate ganglion (see [26,27]).

Temporal evolution of taste responses across an ensemble has also been reported for insular cortex [4] but the nature of the process appears distinct from the PBN. In cortex, neural firing rates are hypothesized to first represent somatosensory aspects of the stimulus, then taste quality, and finally hedonic valence, whereas in our PBN data, these particular shifts were not apparent. An initial representation of somatosensory stimuli by PBN gustatory neurons may well occur, however, since orotactile representation has been widely reported in this region [28,29] and we adapted the mouth to water flow prior to the gustatory stimulus to reduce or eliminate phasic somatosensory signals at taste onset. On the other hand, the successive representation of quality and hedonic value does not appear to take place in the brainstem. This is particularly evident when considering the changes in quinine and cycloheximide representation over time. These stimuli are qualitatively and hedonically similar, i.e. both are bitter and avoided, yet quinine initially groups with salts and acids instead of cycloheximide. Moreover, there is no indication that bitters and acids or sucrose and hypotonic NaCl, pairs which are avoided and preferred respectively, become more closely aligned over time as one would expect with hedonic coding. Thus, a delayed hedonic representation may be a forebrain-specific process. However, this conclusion is somewhat tentative due to the fact that the cortical data was from awake, behaving animals [4], whereas the current study used an acute anesthetized preparation. Certainly, many regions of the forebrain, including insular cortex have projections to the PBN (reviewed in [30]) and these influences would likely be more obvious in awake animals where neural firing is not subjected to the suppressive effects of anesthesia. Further studies will be necessary to resolve this issue.

The delayed narrowing of chemosensitive tuning affected response profiles measured at different time points. Profiles derived from summing over the 10s period were intermediate between those from the early and middle portions of the response, but more closely resembled the latter (see Figure 6). Because the multiple trial requirement limited the number of neurons sampled in the current study, we also scrutinized a much larger sample of PBN neurons gathered from a previous study using similar stimuli but delivered only once [11]. Although it is notable that some neurons in the larger sample, particularly in the N-best group, were narrowly tuned even during the initial response, a similar overall trend for broader initial profiles was apparent. The fact that response profiles can be so broad initially suggests that the degree to which neuron types are apparent with rate-based measures is likely to depend upon the response period analyzed, with clearer types emerging and remaining stable later in the response. Our results also suggest, however, that the notion of chemosensitive neuron types is not purely dependent upon rate since there was a significant interaction between stimulus and neuron type for the response envelope as it unfolded over time. A number of recent neurophysiological studies have concentrated on analyzing the initial response period (e.g., [4,6,8,9,31,32,33]) at times downplaying the concept of chemosensitive neuron types. Recent studies suggest that short periods of time may be sufficient for certain behavioral discriminations, warranting increased focus on the initial time period, but behavioral studies also make it clear that gustatory sensation is persistent and thus later, more stable response periods must be analyzed as well. Both epochs undoubtedly convey gustatory signals that impact behavior.

### Within-Neuron Contribution of Timing

The metric space analysis demonstrated that the temporal aspects of firing augmented quality coding by single neurons more strongly in the initial 1-2s compared to a 2s period in the middle of the response, i.e., the contribution of time was greater during the dynamic, rapidly changing period, when neurons are most broadly tuned. These findings complement earlier observations demonstrating that temporal factors are more important in predicting taste quality for broadly- tuned versus narrowly-tuned neurons in both NST and PBN [5,9]. Our findings imply that temporal features have the potential to help resolve ambiguities in quality coding as expressed in the early relative pattern of firing rates read across the population. By comparing the original data to an exchange-resampled data set, the metric space analysis indicated that the temporal feature making the greatest contribution to between-quality discrimination was the rate envelope. Indeed, in the first 2s, precise spike timing contributed to *H*_max_ in only 10.3% of neurons. These findings are somewhat divergent from earlier studies which suggested that about half of the neurons in both the NST [5] and PBN [9] show some contribution of precise spike timing during this time period. This may be due in part to methodological differences, such as barbiturate vs. urethane anesthesia, or to the fact that the latency between valve opening and stimulus contact in our study was more variable than that reported by Di Lorenzo and Victor [5]. The current results are, however, more consistent with a recent NST investigation in awake, behaving rats that also suggests a more modest contribution from precise spike timing [34]. In any case, our results bolster previous data suggesting that rate envelope can contribute information in both the NST [5] and PBN [9]. Moreover, Figure 5 specifies striking distinctions in response time course that could serve as candidate codes, including differences in latency and the initial rate of rise to the peak response, and even more obviously, the rate and degree of fall in firing rate post-peak.

### Between-Quality Discrimination

**Figure 12:**
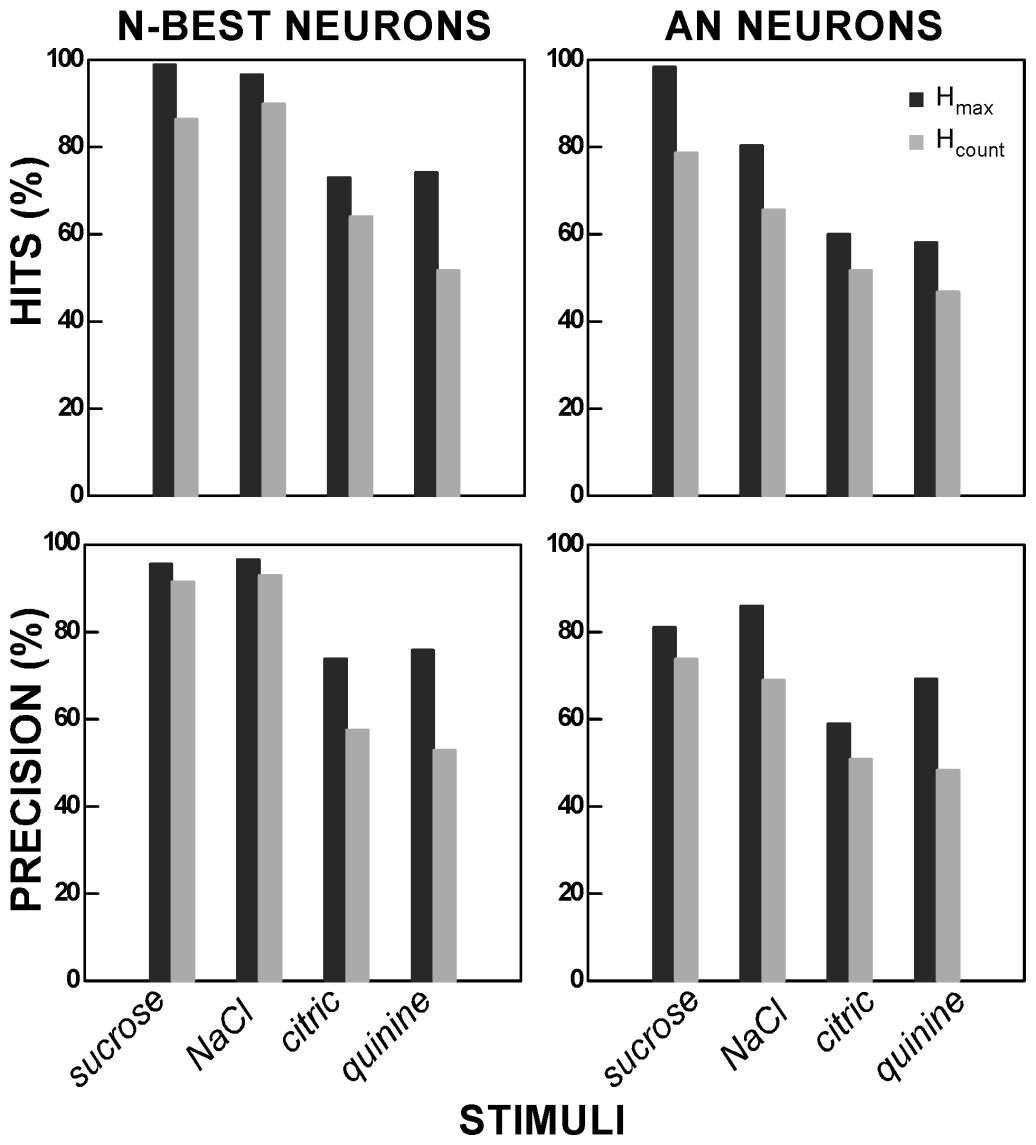
Hit rate and precision for H_count_ and H_max_ during the first second. Hit rate (# stimulus A predictions/# stimulus A presentations) and precision (# correct predictions for stimulus A /# predictions for stimulus A) calculated for *H*_count_ and *H*_max_ from N-best (11 neurons, 88-89 trials for each stimulus) and AN neurons (8 neurons, 60-62 trials for each stimulus) using each of the four representative taste qualities. Although nominal improvements can be seen for both hit rate and precision for each quality, substantial ambiguity remained (e.g., at *H*_max_, the hit rate and precision were quite low for citric acid, especially in AN neurons).

Using a rate-based code, our data suggest that a clear representation of these four distinctive qualities does not emerge until several hundred ms after stimulus onset. On the other hand, behavioral studies have given rise to a general consensus that rats can make taste discriminations very quickly, with some estimates of the minimum time required being as low as 150-200 ms, i.e., 1 lick [35,36,37]. Other estimates are somewhat longer (500-600ms, 4-5 licks [38]). Thus, the time necessary for developing a clear representation based on firing rate seems more protracted in our study than has been indicated by the behavioral data. Whether or not temporal factors alone can fully resolve the mismatch, however, seems unclear. During the first second after stimulus onset, NaCl, citric acid, and quinine elicited robust responses in N-best and AN neurons and minimal responses in S- or B-best cells, suggesting that temporal factors would be required for disambiguation (Figure 6). To obtain a more concrete idea of the impact of temporal factors in resolving this early ambiguity, we used the confusion matrices generated by the STA toolkit to calculate the hit rate (# stimulus “A” predictions/# stimulus “A” presentations) and precision (# stimulus “A” correct predictions/# stimulus “A” predictions) summing across all trials for N-best or AN neurons during the first second. Figure 12 suggests that temporal factors consistently improved both measures but that substantial ambiguity remained.

A major consideration when comparing the current study with the recent taste discrimination literature is the use of anesthetized rats. Although there are neurophysiological data showing that a subset of PBN taste responses can have slow onsets, even in behaving animals [39], an anesthetized, immobile preparation is admittedly a stark contrast to an awake rat, where several variables, including active licking, motivational state, and training, are likely to accelerate responses. In fact, barbiturate anesthesia, such as we used in the present study, is known to suppress neural firing rates [40], in part by potentiating GABA_A_ receptors [41]. However, awake rats may also base discriminations in part on extraneous factors like retronasal and somatosensory cues, or possible fluctuations in gustatory intensity over time, like those suggested by the differences in rate envelope across stimuli observed in the current study. Moreover, while contact with the stimulus delivery spout may be short, in many behavioral paradigms rinses do not occur immediately [35,36,38] leaving the taste stimulus to continue to inform discrimination beyond the contact period. Thus, although the current data may overestimate the time needed for the emergence of an effective ensemble code, existing behavioral data may likewise underestimate the time required for distinctive perceptual representations of stimulus quality to form.

While animal studies have focused on taste perception during the initial phasic period, experiments with human subjects show that taste sensation can fluctuate over time in a manner that varies between compounds and qualities. Many artificial sweeteners, while described as “sweet” when first presented, produce a bitter aftertaste [42]. Unlike “saltiness” which comes on and fades quickly, “bitterness” seems to grow in intensity and lingers even after the taste trial is over (see [43,44]), characteristics that mirror the time course of our B-best neurons. Human subjects also have longer reaction times for bitter and even sweet stimuli than salts, suggesting different neural latencies [45,46,47]. In rodents, stereotyped oromotor behaviors associated with bitter tastants (i.e. gapes) do not begin immediately when the stimulus is delivered, but instead develop slowly, with licking that transitions into gaping [48]. Hence, the full intensity of perceived taste qualities, particularly “bitterness”, may take time to develop.

### Representation of Bitter Taste

In addition to similarities across bitter stimuli, we also found distinguishing neural characteristics between the two bitter stimuli. Consistent with earlier observations, the ionic bitter stimulus, quinine, not only activated B-best cells but also stimulated N- best and AN neurons [11,13]. In the current study, quinine activated electrolyte-sensitive cells more robustly than in the earlier studies, presumably because we deliberately used a log-step higher concentration for just this purpose. Nevertheless, although intense quinine activated multiple neuron types, the response envelopes were dramatically different for B-best versus N-best and AN cells. In the more selective neurons, the rise to the peak response took multiple seconds and was persistent, whereas responses in electrolyte-sensitive neurons peaked in under a second, but adapted to much smaller tonic responses (see Figure 5). Again, compatible with our earlier studies, cycloheximide elicited almost no response in electrolyte-sensitive cells, but was quite effective in B-best neurons. Moreover, although the mean time course for cycloheximide in B-best cells generally resembled that for quinine, it peaked even more slowly. When metric space analysis was applied specifically to bitter responses in B-best cells to test within-quality discrimination, the increase in information afforded by including temporal considerations was dramatic and significant across the population. Because the exchange analysis indicated that rate envelope, not precise spike timing, was the critical variable, it seems plausible that the differences in response onset and time to peak evident in the average rate envelopes at least partially reflect the critical temporal aspects of these responses contributing to increased information.

### Bursting

Both rhythmic and irregular bursting have been observed in a variety of peripheral and central sensory neurons. In fact, oscillatory bursting is particularly evident in another chemosensory modality, olfaction (e.g., [49]). Although not as pervasive, bursting has also been observed in taste-responsive neurons, most obviously in peripheral fibers optimally responsive to sweet stimuli. In these peripheral fibers, sugars and other artificial sweeteners elicit fairly regular rhythmic patterns with a burst frequency of 1-2 Hz [1,2,50,51,52] but this pattern of responsiveness has not been as obvious centrally [53,54]. In the present sample of PBN neurons, bursting was preferentially associated with another class of tastants, bitter stimuli. Although each stimulus elicited some degree of bursting (see Figure 11), this firing pattern was significantly more pronounced for bitter responses, especially in B-best neurons. The bursts were also longer in these cells. These characteristics further delineate B-best cells as a distinct group, and suggest that bursting has some preferential association with “bitterness” or perhaps hedonically aversive stimuli in general. Notably, similar activity has been observed in the GL nerve in response to quinine [55], but has not been reported in quinine- or acid-responsive units of the chorda tympani. Bursting has also been reported in the basolateral amygdala and its projections following conditioned taste and odor aversion [56,57], suggesting that it may be a common feature of responses to aversive or particularly salient stimuli. In a more general context, it has been argued that bursts of firing facilitate the reliability of synaptic transmission [58]. It has also been demonstrated that tuning curves generated from spikes in bursts can peak in response to different stimulus features than either the full spike train or isolated spikes, indicative of multiplexing [59,60]. Whether bursting activity in the PBN contributes to similar functions in the taste system is unknown.

### Concluding Remarks

The current data provide compelling evidence supporting previous findings of systematic temporal variation in gustatory responses. The metric space analysis demonstrates that most individual PBN neurons exhibit consistent differences in temporal dynamics that can lead to more reliable between- or within-quality discriminations, albeit to varying degrees. In the present study, these temporal distinctions were based largely on rate envelope and restricted primarily to the first part of the response. Rate coding was sufficient and stable for many seconds thereafter. The fine-grained analysis of response time course implies that the critical differences in rate envelope include latency and rate of rise to (and fall from) the peak response, characteristics that are a function of both the stimulus and neuron type. A reasonable hypothesis is that these differences reflect the dynamics of the interaction between the stimulus and receptor as well as subsequent processing between and within taste buds. Central processing undoubtedly affects temporal properties as well, although the PBN response envelopes generally resembled those collected with similar procedures in NST (data not shown) supporting the idea that at least some of the temporal features arise upstream, perhaps even in the periphery. One plausible consequence of these temporal dynamics is that they contribute directly to quality coding. However, given their features, it seems equally likely that these temporal variations impart intrinsic secondary characteristics to a given stimulus that are perceived as detectable differences in the rate at which the intensity of sensation grows or declines over time. Taste quality, intensity, and temporal characteristics each provide salient cues that can aid animals in the important task of dietary choice.
